# Products of Chemoenzymatic Synthesis Representing MUC1 Tandem Repeat Unit with T-, ST- or STn-antigen Revealed Distinct Specificities of Anti-MUC1 Antibodies

**DOI:** 10.1038/s41598-019-53052-1

**Published:** 2019-11-12

**Authors:** Yayoi Yoshimura, Kaori Denda-Nagai, Yoshie Takahashi, Izuru Nagashima, Hiroki Shimizu, Toshimitsu Kishimoto, Miki Noji, Shigeyuki Shichino, Yasunori Chiba, Tatsuro Irimura

**Affiliations:** 1 Japan Bioindustry Association, 2-26-9 Hatchobori, Chuo-ku, Tokyo 104-0032 Japan; 2 Biotechnology Research Institute for Drug Discovery, Department of Life Science and Biotechnology, Advanced Industrial Science and Technology (AIST), 1-1-1 Umezono, Tsukuba, Ibaraki 305-8568 Japan; 3 Division of Glycobiologics, Intractable Disease Research Center, Graduate School of Medicine, Juntendo University, 2-1-1 Hongo, Bunkyo-ku, Tokyo 113-8421 Japan; 4 Bioproduction Research Institute, Department of Life Science and Biotechnology, Advanced Industrial Science and Technology (AIST), 1-1-1 Higashi, Tsukuba, Ibaraki 305-8568 Japan; 5 Division of Molecular Regulation of Inflammatory and Immune Diseases, Research Institute of Biomedical Sciences, Tokyo University of Science, 2641 Yamasaki, Noda, Chiba, 278-0022 Japan

**Keywords:** Drug development, Drug development

## Abstract

Anti-mucin1 (MUC1) antibodies have long been used clinically in cancer diagnosis and therapy and specific bindings of some of them are known to be dependent on the differential glycosylation of MUC1. However, a systematic comparison of the binding specificities of anti-MUC1 antibodies was not previously conducted. Here, a total of 20 glycopeptides including the tandem repeat unit of MUC1, APPAHGVTSAPDTRPAPGSTAPPAHGV with GalNAc (Tn-antigen), Galβ1-3GalNAc (T-antigen), NeuAcα2-3Galβ1-3GalNAc (sialyl-T-antigen), or NeuAcα2-6GalNAc (sialyl-Tn-antigen) at each threonine or serine residue were prepared by a combination of chemical glycopeptide synthesis and enzymatic extension of carbohydrate chains. These glycopeptides were tested by the enzyme-linked immunosorbent assay (ELISA) for their capacity to bind 13 monoclonal antibodies (mAbs) known to be specific for MUC1. The results indicated that anti-MUC1 mAbs have diverse specificities but can be classified into a few characteristic groups based on their binding pattern toward glycopeptides in some cases having a specific glycan at unique glycosylation sites. Because the clinical significance of some of these antibodies was already established, the structural features identified by these antibodies as revealed in the present study should provide useful information relevant to their further clinical use and the biological understanding of MUC1.

## Introduction

Mucin 1 (MUC1) was discovered as a carcinoma-associated mucin-like glycoprotein antigen and a mucin representing the major high-molecular-weight and peanut agglutinin-reactive glycoprotein^1^. It has hallmarks of membrane-associated mucins, such as an extracellular domain with threonine-rich and serine-rich tandem repeats of 20 amino acids (APPAHGVTSAPDTRPAPGST), a self-catalytic neck domain, a transmembrane domain, and an interaction-prone cytoplasmic tail with many tyrosine residues. MUC1 is ubiquitous among all epithelia and is apparently released into stromal tissue and circulation under disease conditions, whereas it covers the luminal sides of normal epithelia. It is believed that the attachment density and the structure of *O*-glycans of MUC1 expressed by carcinoma cells are distinct from those of normal epithelia-associated MUC1. By this and by many other reasons, MUC1 is considered as the prime target of specific cancer immunotherapy and various forms of vaccines were designed and tested clinically and pre-clinically^2^.

Many monoclonal antibodies (mAbs) specific for MUC1 were developed and some of them resulted from immunisation with carcinoma cells and screening for the binding to carcinoma cells. After the gene for the MUC1 core polypeptide was identified and purified, recombinant or synthetic glycopeptides were often used for immunisations and antibody screenings. Some of anti-MUC1 mAbs, for instance 115D8, are marketed world-wide and have been used as a standard clinical tool to monitor tumour burden in breast cancer for nearly 30 years (CA15-3)^3^, even though the precise epitope structure in the MUC1 detected by the antibody remains unknown. Another mAb, KL-6, has been used as a serum marker for interstitial pneumonia^4^.

Increasing evidence supports a notion that reactivity of many anti-MUC1 mAbs depends on MUC1 glycosylation to varying extents. Karsten and co-workers prepared synthetic glycopeptides with attached Tn antigen (GalNAc) or T (also sometimes called as TF) antigen (Galβ1-3GalNAc) and used them to compare the specificity of anti-MUC1 mAbs^5^. They found that many anti-MUC1 mAbs recognise the peptide sequence around APDTR, and interestingly many of the antibodies showed higher affinity to the glycopeptide having Tn or T epitopes on its threonine residue than to its unglycosylated counterparts. The length of polypeptide representing the number of tandem repeats also strongly contributes to the binding of some anti-MUC1 antibodies^6^. Other studies with synthetic glycopeptides by Tarp and co-workers^7^, Ohyabu and co-workers^8^, and Rangappa and co-workers^9^ revealed glycan structures required for the recognition by a limited number of anti-MUC1 antibodies but did not reach a systematic understanding and classification of these antibodies. Zhou and co-workers attempted to classify anti-MUC1 mAbs based on the nature of their epitopes mostly based on previously published observations^10^.

In the present report, we have used a systematic approach to extend and deepen such classification. We prepared a series of 20 glycopeptides, each having one of four different carbohydrate chains, Tn-, T-, sialyl-T (ST)-, and sialyl-Tn (STn)-antigen, at one of five possible *O*-glycosylation sites on a 27-mer peptide including the tandem repeat unit of MUC1. T-, ST-, and STn-MUC1 glycopeptides were synthesised from Tn-MUC1 glycopeptides in preparative scale by utilising three glycosyltransferases expressed by a methylotrophic yeast, *Ogataea minuta*. The glycopeptides were tested for their binding to 13 anti-MUC1 mAbs. Using this approach, we were able to classify these antibodies according to their specificity for carbohydrate-protein complexes including the attachment positions. The results could be useful to interpret the differential characteristics of anti-MUC1 mAbs currently in use as diagnostic or therapeutic tools.

## Results

### Preparation of glycopeptides with a single *N*-acetylgalactosamine (GalNAc) residue attached to Thr or Ser residue of the tandem repeat structure of MUC1

**Figure 1:**
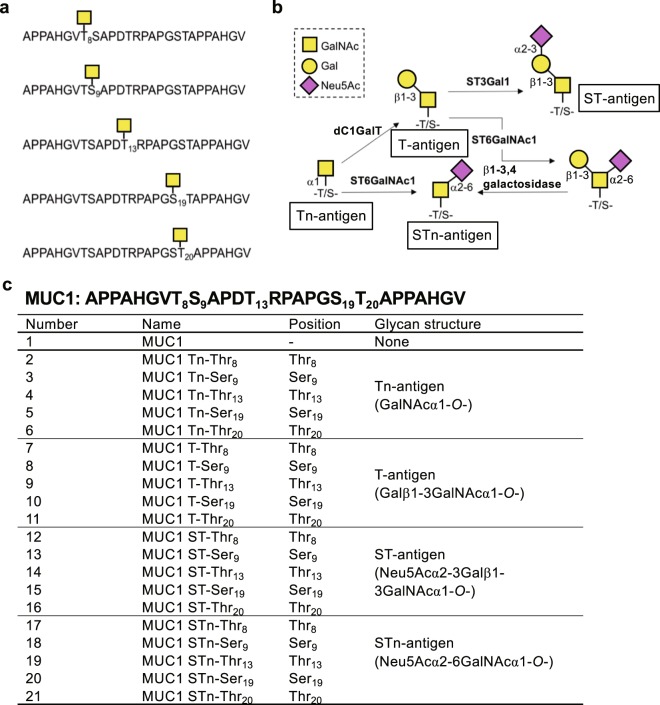
The structures of glycopeptides and glycans. (**a**) Chemically synthesised MUC1 GalNAc glycopeptides, (**b**) schematic explanation of the potential enzymatic synthesis pathway of *O*-glycopeptides and (**c**) list of MUC1 glycopeptides synthesised in this study.

We prepared glycopeptides containing GalNAc residues attached to one of three Thr or two Ser residues of a 27-mer peptide APPAHGVTSAPDTRPAPGSTAPPAHGV including the 20-mer tandem repeat unit of MUC1. The last seven residues APPAHGV are the same as the first seven residues allowing to form an antigenic epitope on either side of the glycans (Fig. 1a). The five GalNAc-containing glycopeptides in Fig. 1a were chemically synthesised by conventional 9-fluorenylmethoxycarbonyl group-solid-phase peptide synthesis (Fmoc-SPPS) under microwave irradiation^11^.

### Enzymatic reactions on MUC1 glycopeptides and evaluation of substrate preferences of drosophila core 1 synthase (dC1GalT), beta-galactoside alpha-2,3-sialyltransferase 1 (ST3Gal1) and *N*-acetylgalactosaminide alpha-2,6-sialyltransferase 1 (ST6GalNAc1)

Using the synthesised GalNAc-containing glycopeptides, we extended their carbohydrate portions to carry T-, ST-, or STn-antigen at each Thr or Ser residue using the purified enzymes (Fig. 1b,c).

**Figure 2:**
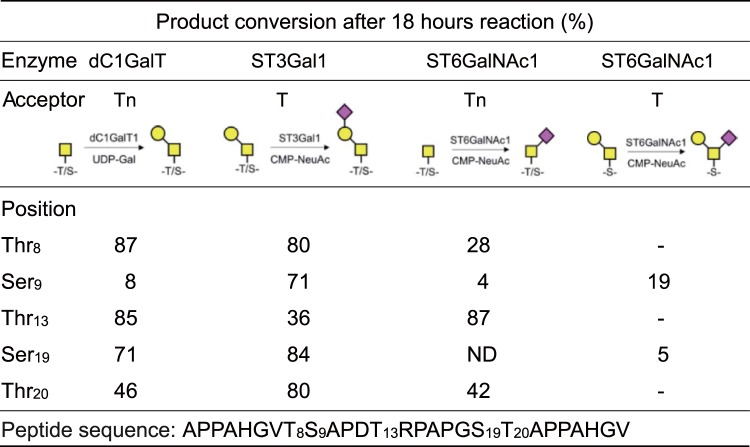
Relative substrate-product conversion rates of *O*-linked carbohydrate chains by glycosyltransferases after 18 hours reaction. Before preparative-scale syntheses of glycopeptides, we checked the reactivity of each glycosyltransferase toward different substrates by this substrate-product conversion assay. Each enzyme was tested with glycopeptides whose sequence and glycan attachment sites are shown below. The structures of the glycans before and after conversion by the enzymes are shown. Percentages indicate the relative area under the curve of the HPLC peak of the product as compared to 100% conversion. Data shown are the means of two independent experiments. ND, not detected; -, not done.

dC1GalT was used for preparations of T-antigen structure on glycopeptides with a GalNAc residue. To investigate substrate preference, diluted dC1GalT was used at small-scale reaction. After three-hour incubation, dC1GalT converted more than 40% of the GalNAc glycopeptides into galactosylated products in three of five substrates. The conversion rate after 18 hours was found to be in the following order: MUC1 Tn-Thr_8_ (87%) ≒ MUC1 Tn-Thr_13_ (85%) > MUC1 Tn-Ser_19_ (71%) > MUC1 Tn-Thr_20_ (46%) ≫ MUC1 Tn-Ser_9_ (8%) (Fig. 2). In all substrates, from six to 18 hours after starting the reaction, product conversion became slow since dC1GalT lost almost half of its activity during the incubation at 37 °C (data not shown). Conversion of MUC1 Tn-Ser_9_ by dC1GalT was 10 times less efficient than that of MUC1 Tn-Thr_8_.

ST3Gal1 was also tested for its activity to convert T-antigen into ST-antigen. The yield after 18 hours reaction was more than 70%, except for MUC1 T-Thr_13_ (Fig. 2). Although the product formation of MUC1 ST-Thr_13_ was two times less than other substrates and the final product conversion was 36% at 18 hours, the differences in the reactivity of ST3Gal1 among the five substrates was not remarkable as observed with dC1GalT.

For the synthesis of STn glycopeptides, recombinant ST6GalNAc1 was applied at first. The amount of ST6GalNAc1 conversion rates of four substrates were: MUC1 Tn-Thr_13_ (87%) > MUC1 Tn-Thr_20_ (42%) > MUC1 Tn-Thr_8_ (28%) ≫ MUC1 Tn-Ser_9_ (4%) (Fig. 2). Product formation was not observed with MUC1 Tn-Ser_19_ after 18 hours incubation (Fig. 2). To assess the effects of amino acid side chains, we also tested ST6GalNAc1 with two modified glycopeptides in which Ser-GalNAc residue was substituted by Thr-GalNAc (MUC1 Tn-Ser_9_ → Thr_9_ and MUC1 Tn-Ser_19_ → Thr_19_). ST6GalNAc1 acted on both Thr-substituted substrates and produced STn-glycopeptides with over 50% yield after incubation for 18 hours (Supplementary Table S1). These results were consistent with a previous report by Blixt and co-workers showing that the chicken ST6GalNAc1 showed a lower activity to GalNAc-Ser residue compared to GalNAc-Thr^12^. To prepare STn at Ser_19_, we used MUC1 T-Ser_9_ and MUC1 T-Ser_19_ as the substrate of ST6GalNAc1, because mouse ST6GalNAc1 was previously reported to prefer T-antigen to Tn-antigen structure^13^. As shown in Fig. 2, ST6GalNAc1 reacted on both Ser-T-antigen glycopeptides and gave putative α2,6-sialylated products. On MUC1 T-Ser_9,_ yields by ST6GalNAc1 were 3%, 6%, and 19% after 3, 6 and 18 hours of incubation, respectively. In the case of MUC1 T-Ser_19_, the product was observed only after 18 hours reaction, and the conversion rate was 5%.

### Preparative-scale synthesis of MUC1 glycopeptides bearing T-, ST- or STn structure

To prepare various glycopeptides to be used in the antibody binding assays, preparative-scale reactions (more than 50 μg of each product) with the three glycosyltransferases used in the small-scale experiments were also performed. The structures of the products were confirmed by high performance liquid chromatography (HPLC) and matrix assisted laser desorption ionization - time of flight mass spectrometry (MALDI-TOF MS). In the preparation of five T-antigen glycopeptides, recombinant dC1GalT was added to more than 600 μU/reaction. The yield of targeted glycopeptides was 80–95% (Supplementary Fig. S1). To prepare ST-MUC1 glycopeptides, ST3Gal1 was used under similar conditions as dC1GalT. The yield of each ST glycopeptide was 70–90% (Supplementary Fig. S2).

**Figure 3:**
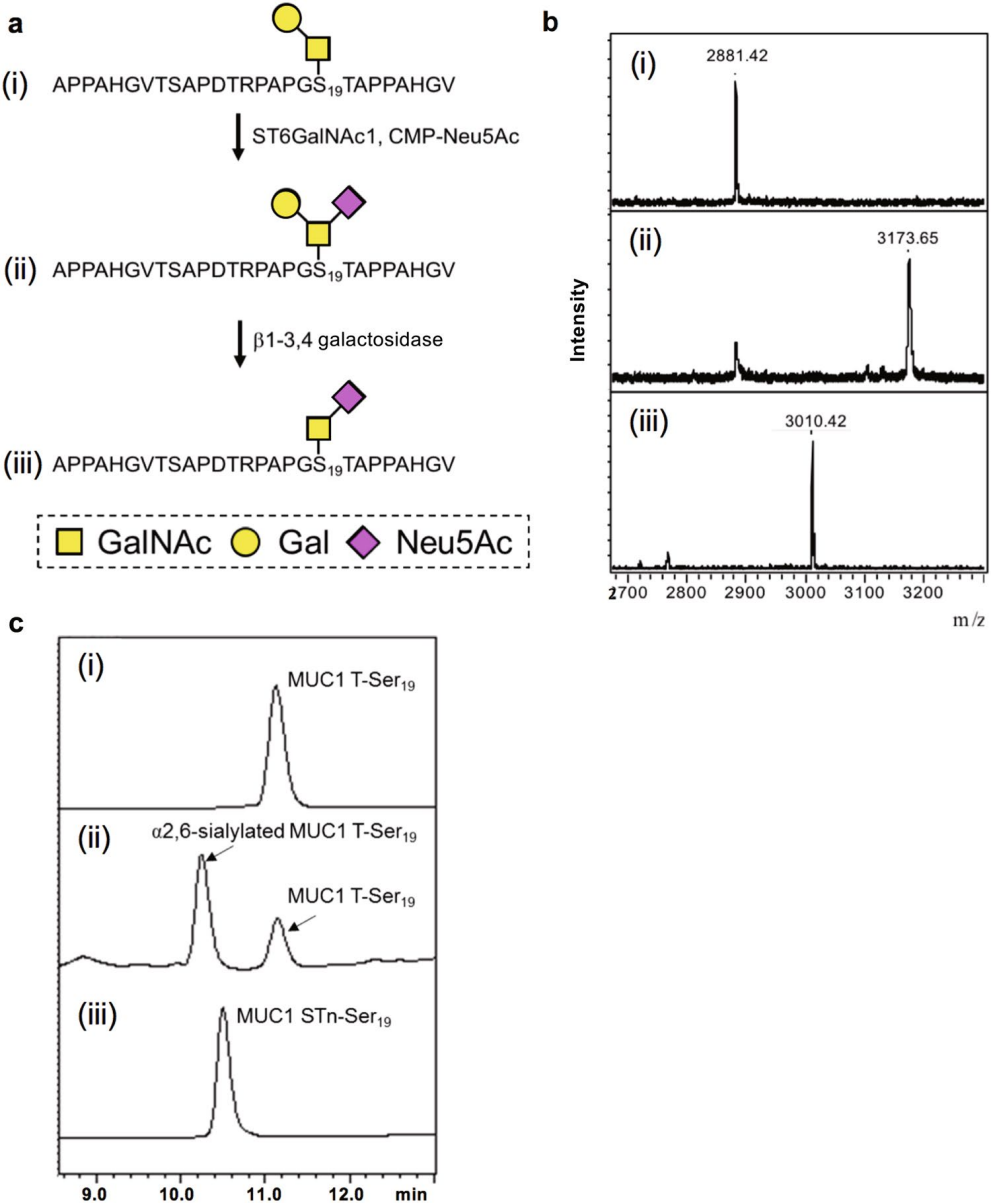
Enzymatic synthesis of MUC1 STn-Ser_19_. (**a**) Schematic representation of the synthesis. (**b**) MALDI-TOF MS analysis. (**c**) HPLC analysis. Within each figure, (i), (ii) and (iii) correspond to MUC1 T-Ser_19_, α2,6-sialylated MUC1 T-Ser_19_ (in reaction mixture) and MUC1 STn-Ser_19_ (in reaction mixture), respectively. The final reaction with β1-3,4 galactosidase was performed on the purified α2,6-sialylated MUC1 T-Ser_19_.

Three STn-glycopeptides (MUC1 STn-Thr_8_, MUC1 STn-Thr_13_ and MUC1 STn-Thr_20_) were synthesised with ST6GalNAc1 from each corresponding Tn glycopeptide in a similar fashion (Supplementary Fig. S3). For the synthesis of MUC1 STn-Ser_9_ and MUC1 STn-Ser_19_, MUC1 T-Ser_9_ and MUC1 T-Ser_19_ were employed as substrates based on the information from the small-scale reactions. Figure 3a shows the route of the synthesis of MUC1 STn-Ser_19_ accomplished by the following two steps. In the first step with ST6GalNAc1, a large amount of the enzyme and further addition of the enzyme during the reaction boosted the sialylation on MUC1 T-Ser_19_ up to a 70% yield. After purifying the sialylated product, the unnecessary galactose residue was removed by commercially available β1-3,4 galactosidase and MUC1 STn-Ser_19_ was produced (Fig. 3b,c). MUC1 STn-Ser_9_ was synthesised in a similar manner as MUC1 STn-Ser_19_ (Supplementary Fig. S4).

**Figure 4:**
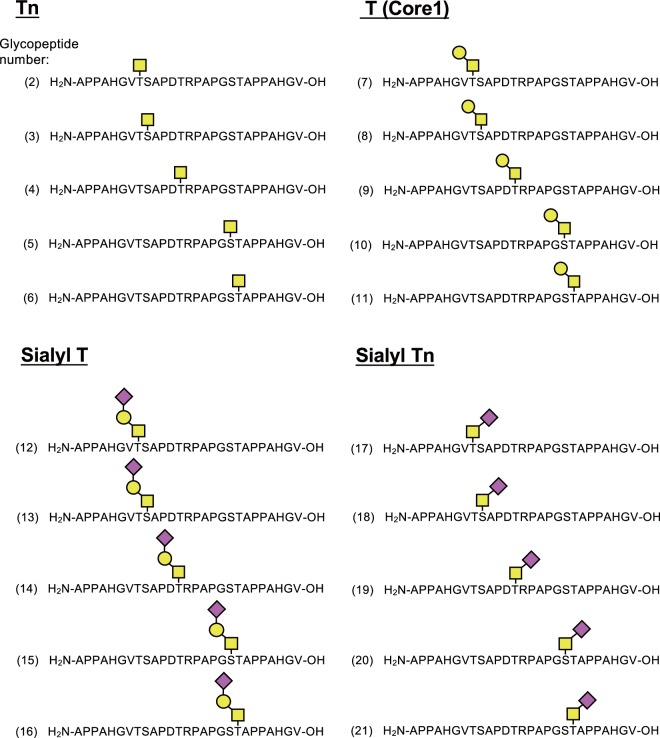
Chemical structures of the 20 glycopeptides as grouped according to the antigen structures. The glycopeptide numbers (in brackets) correspond to the glycopeptide numbers used in Figs 1 and 5.

All glycopeptides synthesised in this study are schematically shown in Fig. 4. Glycopeptide products were purified by HPLC as described in the experimental procedures and their structures were confirmed by MALDI-TOF MS (Supplementary Figs S5–S8).

### Reactivity of 13 anti-MUC1 mAbs with the glycopeptides

**Figure 5:**
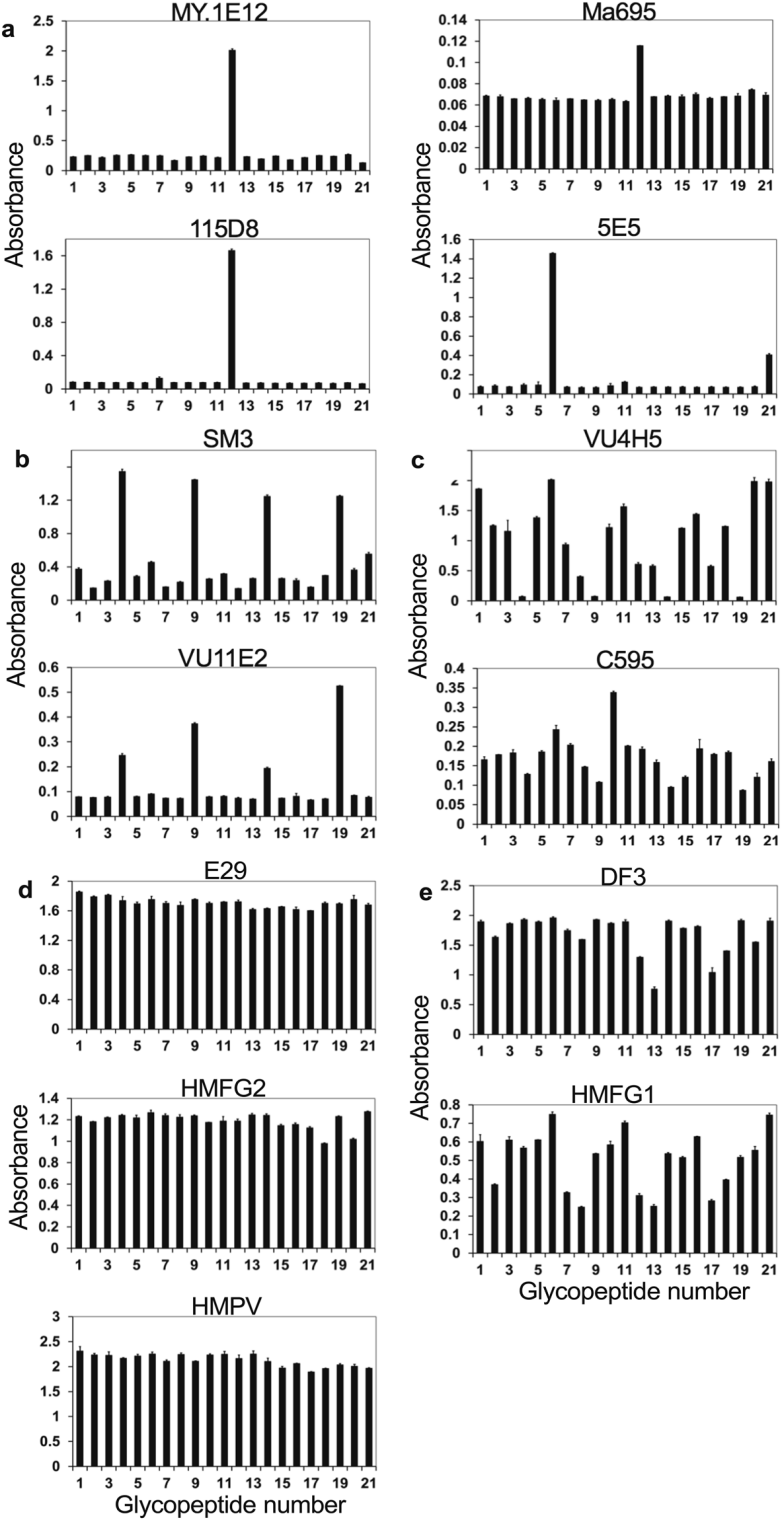
Binding patterns of 13 anti-MUC1 mAbs to a panel of 20 glycopeptides and a naked peptide as investigated by ELISA. The binding patterns can be divided into five groups. (**a**) Group one: antibodies which bind a glycopeptide with a distinct glycan at a distinct amino acid site: these antibodies bind MUC1 27-mer with ST-antigen on Thr_8_ or Tn-/STn-antigen on Thr_20_. (**b**) Group two: antibodies whose bindings increase when a distinct amino acid site is glycosylated: these antibodies bind MUC1 27-mer with any carbohydrate chain extending from Thr_13_. (**c**) Group three: antibodies whose bindings decrease when a distinct amino acid site is glycosylated: these antibodies show low or virtually no binding when Thr_13_ of MUC1 27-mer is glycosylated with any glycan. (**d**) Group four: antibodies whose bindings are glycosylation-independent: antibody binding to MUC1 27-mer is not affected by glycosylation on any of the five glycosylation sites. (**e**) Group five: antibodies with no particular antibody binding patterns as revealed by the comparison of binding profiles to these 20 glycopeptides and a naked 27-mer peptide. The names of the anti-MUC1 mAbs are indicated above the graphs. Data are shown as means ± SD.

Enzyme-linked immunosorbent assay (ELISA) was performed using 96-well microtiter plates. Plate surfaces were coated with the 20 glycopeptides and a peptide without attached carbohydrate chains (naked peptide). Absorbance at 405/492 nm was used as a readout for antibody binding. For each antibody, absorbance readings for the 20 glycopeptides and the naked peptide were plotted into a graph to compare their differential reactivity. As the result, the binding profiles of anti-MUC1 mAbs were found to be highly diverse but the specificities can be classified into distinct groups according to the nature of the structural requirements, as shown in Fig. 5. MY.1E12, 115D8 and Ma695 only bound ST attached to Thr_8_ and are considered to be specific for an epitope formed by a combination of a carbohydrate chain and peptide sequences (Fig. 5a). Pearson’s correlation coefficients between the binding patterns of MY.1E12, 115D8 and Ma695 also revealed a very strong positive correlation (Pearson’s r ≥ 0.97), supporting the similarities in binding specificities of these mAbs (Supplementary Fig. S9). 5E5 bound a glycopeptide with Tn attached to Thr_20_ and weakly to that with STn attached to Thr_20_ (Fig. 5a). Therefore, we classified these mAbs into one group because they were specific for epitopes formed by combinations of a carbohydrate chain and the peptide sequence or the attachment site. Such specificity toward the unique glycan structure and the attachment site has not previously been shown, except that it was previously suggested for MY.1E12 (ref.^14^). Antibodies in the second group, SM3 and VU11E2, strongly bound to glycopeptides with an attached carbohydrate chain at Thr_13_, regardless of the structure (Fig. 5b). Antibodies in the third group, VU4H5 and C595, were reactive with almost all glycopeptides, though their reactivity was low or virtually none when a carbohydrate chain is attached at Thr_13_ (Fig. 5c). Antibodies in the fourth group, E29, HMFG2, and HMPV, bound to all glycopeptides and the naked peptide regardless of the attachment of carbohydrate chains (Fig. 5d). The specificities of the antibodies in the fifth group, DF3 and HMFG1, did not follow any observable pattern (Fig. 5e) as far as their reactivity with these 20 glycopeptides and the naked peptide was compared. Pearson’s correlation coefficients between binding patterns of the mAbs also revealed strong to moderate positive correlations (Pearson’s r ≥ 0.44–0.97) within the second, the third and the fifth group, supporting the similarities in binding specificities of the mAbs within these groups (Supplementary Fig. S9).

## Discussion

In the present study, we hypothesised that some of the available anti-MUC1 mAbs are likely to recognise epitopes formed by combinations of glycans and backbone peptides in the MUC1 tandem repeat and hence we decided to evaluate their specificity systematically by synthesising 20 glycopeptides with four structurally defined single *O*-glycans attached at different sites. The results revealed novel details on the substrate specificity of dC1GalT and ST6GalNAc1, which were used in the glycopeptide synthesis to extend the carbohydrate chains beyond GalNAc. We used these 20 glycopeptides to investigate the binding specificities of 13 anti-MUC1 mAbs. Some of the antibody specificities were previously unknown. This is the first report to show clearly that specificities of anti-MUC1 mAbs are diverse, and that they can be classified into at least five distinct groups. Validity of the grouping was revealed by the Pearson’s correlation analysis.

Although it is widely known that polypeptide GalNAc-transferases (ppGalNAc-Ts), an enzyme family catalysing the initial steps in mucin-type glycan synthesis, have different substrate specificities depending on the positions of Ser/Thr residues in glycopeptides^15^, the substrate preference of glycosyltransferases involved in the subsequent extension of *O*-glycans is poorly investigated. In the course of the present study, we found that dC1GalT and ST6GalNAc1 showed variable activity according to the position of the GalNAc residue in the peptide sequence and to the side residue of the amino acid (*i.e*. Ser or Thr) within the MUC1 sequence. This difference may be at least in part due to conformational changes of the peptide backbone after GalNAc incorporation as observed with Thr_13_ of MUC1 (ref.^16^). We found that sialic acid was not efficiently transferred by ST6GalNAc1 directly to GalNAc residues attached to Ser_9_ or Ser_19_ of the MUC1 glycopeptide used in the present study. This information was essential to designing optimal conditions for the large-scale preparation of all necessary glycopeptides. From our results, it is clear that the structural extension of *O*-glycans is not a random but rather a highly regulated process whose efficiency is decided by the position and the type of amino acid even in the abundant presence of glycosyltransferases. MUC1 is expressed by almost all epithelia, by carcinomas, and (at low levels) by other types of cells, and the extent and the profile of glycosylation is likely to be unique to the particular type of cell. Understanding the patterns of extension of MUC1 glycoforms at a particular target site will be an important and challenging issue in optimizing the specific, goal-oriented use of various anti-MUC1 mAbs.

According to our results, specificities of anti-MUC1 mAbs can be classified into five groups and the grouping was statistically supported by the Pearson’s correlation analysis. The epitopes for mAbs in the first group should be formed by a combination of a carbohydrate chain and peptide sequences. The importance of ST structure attached to Thr_8_ for MY.1E12 was previously suggested^14^, but this was clearly demonstrated by the present investigations. Also, from the results of the present study it became clear that the specificities of 115D8, Ma695, and MY.1E12 are very similar. The epitope for 5E5 is also formed by a combination of Tn attached to Thr_20_ and weakly cross-reactive with STn attached to Thr_20_. The preference of 5E5 binding to MUC1 with Tn and STn attached to Thr_20_ was not previously known^7^. Because of the nature of the epitope, we classify 5E5 together with MY.1E12, 115D8 and Ma695 into one group.

Antibodies in the second group, SM3 and VU11E2, are considered to be specific for an epitope formed by the peptide and its accessibility seems to be enhanced by glycosylation of Thr_13_ present in the PDTR epitope. In support of our results, it was previously shown that GalNAc introduction at the Thr in the PDTRP motif of synthetic MUC1 peptides increases the binding affinities of SM3 and VU11E2 by a mechanism that stabilizes the conformation of the binding epitope without altering the peptide contact sites, though the contribution of some GalNAc residues to the binding was suggested^17,18^.

Antibodies in the third group, VU4H5 and C595 are reactive with almost all glycopeptides but the reactivity is low or virtually none when a carbohydrate chain is present at Thr_13_. Therefore, they recognise a similar epitope to that of antibodies in the second group but the accessibility is reduced when Thr_13_ is glycosylated. In fact, the peptide epitopes of VU4H5 (APDTR) and VU11E2 (TSAPDTR) have been shown to be very similar^19^, but our data show that the absence or presence of a glycan on Thr_13_ differentially affects their bindings. The difference in immunogens used for the preparation of VU4H5 (a MUC1 60-mer peptide conjugated to bovine serum albumin (BSA)) and VU11E2 (ZR75-1 breast cancer cells)^19^ may have influenced their epitope.

Antibodies in the fourth group, E29, HMFG2 and HMPV, bind to glycopeptides and naked peptide regardless of the attachment of carbohydrate chains indicating that the epitope is a peptide and the binding is not affected by the glycosylation of any Thr or Ser when a single glycan is attached.

This classification will be useful to distinguish differentially glycosylated MUC1 biologically relevant to health and disease, such as MUC1 expressed by breast cancer tissue and normal ductal epithelia, and to correlate the differential bindings to the expression profiles of glycosyltransferases. As an example, Thr_13_ is known to be glycosylated by ppGalNAc-T4 (refs^20,21^) and possibly other specific ppGalNAc-Ts. Therefore, formation of the epitope reactive with anti-MUC1 antibodies in group 2 and 3 should also be dependent on the expression of this unique group of ppGalNAc-Ts. On the other hand, GalNAc attachment to Thr_8_ seems to be catalyzed by various ppGalNAc-Ts^22,23^, but the glycan extension to form ST at this particular site may require specific glycosyltransferases. Studies considering the relationship between anti-MUC1 antibody binding and the expression of glycosyltransferases will open a new perspective on the use of various anti-MUC1 mAbs in the near future.

Although studies with synthetic glycopeptides representing portions of MUC1 tandem repeats were conducted previously, systematic understandings were not previously reached. For example, 5E5 was shown to have high specificity to Tn-MUC1 and STn-MUC1 (ref.^24^), but the present result is the first to show that the attachment site should be Thr_20_. The difficulty in preparing *O*-linked glycopeptides with the full spectrum of structural variations might have previously hindered clear understanding of the antibody specificity, which was overcome in the present study. Thus, a previous attempt by Zhou and co-workers to classify anti-MUC1 mAbs based on the nature of their epitope was not complete because they did not consider which Thr/Ser residue was glycosylated^10^.

The findings of the present study have direct implications for the development of improved MUC1-based tools for the diagnosis and therapy of breast and other types of cancer, considering that a mAb explored in the present study, 115D8, is widely used to detect serum biomarker CA15-3, and that 5E5, SM3 and HMFG2 are used to develop chimeric antigen receptor T-cells^25,26^. Therefore, the present findings should pave the way for the development of new anti-MUC1 mAbs with improved specificities to advance future cancer therapy.

## Materials and Methods

### General

Reagents were purchased from Fujifilm Wako Pure Chemical Corp. (Osaka, Japan) and Sigma-Aldrich (St. Louis, MO), and used without further purification. MUC1 glycopeptides were synthesised by Fmoc-SPPS using Fmoc-Val preloaded NovaSyn TGA resin (Novabiochem, Darmstadt, Germany), Fmoc-amino acids (Novabiochem): Fmoc-Ala-OH, Fmoc-Arg(Pbf)-OH, Fmoc-Asp(O*t*Bu)-OH, Fmoc-Gly-OH, Fmoc-His(Trt)-OH, Fmoc-Pro-OH, Fmoc-Ser(*t*Bu)-OH, Fmoc-Thr(*t*Bu)-OH or Fmoc-Val-OH, and glycosylated Fmoc-amino acids (Medicinal Chemistry Pharmaceutical, Sapporo, Japan): Fmoc-Thr(Ac_3_GalNAcβ1→)-OH or Fmoc-Ser(Ac_3_GalNAcβ1→)-OH. Coupling of glycosylated Fmoc-amino acids was performed by a microwave synthesiser, Wave Magic MWS-1000A (EYELA, Tokyo, Japan). MALDI-TOF MS spectra were recorded on an Ultraflex (Bruker Daltonik GmbH, Bremen, Germany) in linear positive ion mode with 2.5-dihydroxybenzoic acid (DHB, 10 mg/mL in 30% acetonitrile solution containing 0.1% trifluoroacetic acid) as the matrix. Protein purification was performed at 4 °C on an AKTAexplorer 10 S system (GE Healthcare, Buckinghamshire, UK).

### Antibodies

The following anti-MUC1 mAbs were used: 115D8 (Abcam, Cambridge, UK), HMPV (BD Pharmingen, San Diego, CA), Ma695 (Fujirebio, Tokyo, Japan), HMFG1 (aka 1.10.F3) (Abcam), VU4H5 (Santa Cruz Biotechnologies, Dallas, TX), VU11E2 (GeneTex, Irvine, CA), SM3 (Abcam), C595 (NCRC48) (Abcam), E29 (Abcam), HMFG2 (Millipore, Burlington, MA), DF3 (LSBio, Seattle, WA). MY.1E12 was previously established by our group^27^, and 5E5 was previously established and kindly provided by Dr. H. Clausen^24^. The following isotype control antibodies were purchased: purified mouse IgG1κ, purified mouse IgG2a, and purified mouse IgG2b (BioLegend, San Diego, CA).

### Synthesis of GalNAc glycopeptides by Fmoc-SPPS

Fmoc-Val-NovaSyn TGA resin (0.26 mmol/g, 100 mg, 0.026 mmol) in a LibraTube® (5 mL capacity) was allowed to swell in *N*,*N*-dimethylformamide (DMF) for a period of two hours. To remove Fmoc group, 20% piperidine in DMF (2 mL) was added to the filtered resin and the mixture was shaken for five minutes at 50 °C. After the resin was washed by DMF, the coupling reaction was performed. Couplings of the corresponding Fmoc-amino acid (0.13 mmol, 5.0 eq.) were performed by shaking at 50 °C with 1-[(1-(Cyano-2-ethoxy-2-oxoethylideneaminooxy) dimethylaminomorpholino)] uronium hexafluorophosphate (COMU) (0.13 mmol, 5.0 eq.) and *N*,*N*-diisopropylethylamine (DIPEA) (0.13 mmol, 5.0 eq.) in DMF (0.5 mL) for 20 minutes. For glycosylated residues, coupling reactions were performed at 50 °C for 20 minutes with glycosylated Fmoc-amino acids (0.031 mmol, 1.2 eq), COMU (0.031 mmol, 1.2 eq.) and DIPEA (0.031 mmol, 1.2 eq.) in DMF (0.5 mL) under microwave irradiation with Wave Magic MWS-1000A (EYELA). Assuming that unreacted amino groups remained on the resin, the resin was then treated with a mixture of acetyl capping cocktail [acetic anhydride (4.75%, v/v), DIPEA (2.25%, v/v), and 1-hydroxybenzotriazole (HOBt) (13 mM) in DMF (1 mL)] for three minutes at room temperature. These three procedures (deprotection of Fmoc group, coupling of Fmoc-amino acid or glycosylated Fmoc-amino acid, and acetyl capping) were carried out repeatedly 26 times. Only the very last acetyl capping, which was practically not required, was not performed. To complete the syntheses of 27 amino acid residual MUC1s, each resin was treated with 2.5 mL of cleavage cocktail (trifluoroacetic acid:triisopropylsilane:water = 95:2.5:2.5) for two hours at room temperature, filtered, and washed twice with the same cocktail. The combined filtrates were concentrated by streaming of nitrogen gas. The residue was precipitated by addition of cold diethyl ether in an ice bath to give a solid. The solid was washed with cold diethyl ether twice, dried by streaming of nitrogen gas, dissolved in 10% acetonitrile solution and lyophilised. The crude material was purified by a preparative reversed phase-HPLC (column: Inertsil^®^ ODS, Ø 20 mm × 250 mm; column oven temperature: 40 °C; flow rate: 6 mL/minute; eluent A: water with 0.1% trifluoroacetic acid; eluent B: acetonitrile with 0.1% trifluoroacetic acid; detection: 220 nm UV; gradient: 15% B to 40% B over 60 minutes (condition A), 10% B to 40% B over 60 minutes (condition B) or 10% B to 35% B over 60 minutes (condition C)) to give acetylated MUC1 GalNAc glycopeptides: MUC1 Ac_3_Tn-Thr_8_ 34.3 mg (46% yield, HPLC: condition A); MUC1 Ac_3_Tn-Ser_9_ 27.3 mg (37% yield, HPLC: condition B); MUC1 Ac_3_Tn-Thr_13_ 42.5 mg (57% yield, HPLC: condition C); MUC1 Ac_3_Tn-Ser_19_ 39.9 mg (54% yield, HPLC: condition C), MUC1 Ac_3_Tn-Thr_20_ 32.7 mg (44% yield, HPLC: condition C). MALDI-TOF MS: C_123_H_190_N_35_O_43_ [M + H]^+^ calculated (m/z) 2845.38, found (m/z) MUC1 Ac_3_Tn-Thr_8_ 2846.16; MUC1 Ac_3_Tn-Ser_9_ 2846.47; MUC1 Ac_3_Tn-Thr_13_ 2846.52; MUC1 Ac_3_Tn-Ser_19_ 2846.15; MUC1 Ac_3_Tn-Thr_20_ 2846.46.

To each portion of purified acetylated MUC1 GalNAc glycopeptides in methanol (2 mL) drops of 28% sodium methoxide in methanol were added to adjust the pH to be over pH 10. The reaction mixtures were stirred for 30 minutes at room temperature, neutralised by acetic acid and concentrated *in vacuo*. The residue was purified by a preparative reversed phase-HPLC (gradient: 7% B to 30% B over 60 minutes, other conditions were the same as described above.) to give MUC1 GalNAc glycopeptides: MUC1 Tn-Thr_8_ 13.1 mg (quant.); MUC1 Tn-Ser_9_ 8.6 mg (quant.); MUC1 Tn-Thr_13_ 10.9 mg (99% yield); MUC1 Tn-Ser_19_ 9.8 mg (97% yield); MUC1 Tn-Thr_20_ 8.3 mg (82% yield). MALDI-TOF MS: C_117_H_184_N_35_O_40_ [M + H]^+^ calculated (m/z) 2719.34, found (m/z) MUC1 Tn-Thr_8_ 2719.37; MUC1 Tn-Ser_9_ 2719.25; MUC1 Tn-Thr_13_ 2719.19; MUC1 Tn-Ser_19_ 2719.11; MUC1 Tn-Thr_20_ 2719.11.

### Enzyme expressions

Three glycosyltransferases (dC1GalT, ST3Gal1 and ST6GalNAc1) were expressed in the yeast strain *O. minuta* (TK-10-1-2)^28,29^. The amino acid sequences of three enzymes were obtained from the UniProt database [dC1GalT (Q7K237), ST3Gal1 (Q11201), ST6GalNAc1 (Q9NSC7)]. The codon-optimised genes for *O. minuta* encoding those glycosyltransferases whose codons were optimised for *O. minuta* expression system were synthesised starting from Ser42 (Δ41, dC1GalT), Asn27 (Δ26, ST3Gal1) and Pro38 (Δ37, ST6GalNAc1), respectively (Eurofins Genomics, Tokyo, Japan). The synthetic genes were inserted into *Bam*HI site of pEX-K4J1 were digested with *Bam*HI and sub-cloned into an expression vector pOMEA-PA10H, in which the gene encoding PA-tag^30^ and 10 × His-tag sequences are inserted just after an initial methionine codon. The resultant expression vectors were purified, digested by *Not*I and electroporated into *O. minuta* TK 10-1-2 cells.

For protein expression, the transformed cells containing expression constructs for each glycosyltransferase integrated into the genome were inoculated into Yeast Extract-Peptone-Adenine-Dextrose (YPAD) medium (3 mL) and cultivated overnight at 30 °C. The overnight culture was transferred to 150 mL of BMGDY medium (1% yeast extract, 2% peptone, 1.34% yeast nitrogen base without amino acids, 0.2 mg/mL of adenine and 0.1 mg/mL of uracil, 2% glycerol, 0.5% glucose, in 100 mM potassium phosphate buffer (pH 6.0)) and cultivated at 30 °C with continuous shaking (140 rpm). After 60 hours of cultivation, cells were harvested by centrifugation (1,400 × *g*) at room temperature. The collected cells were re-suspended with 100 mL of BMMYC medium (1% yeast extract, 2% peptone, 1.34% yeast nitrogen base w/o amino acids, 0.2 mg/mL of adenine, 0.1 mg/mL of uracil, 2% casamino acid and 1% methanol in 100 mM potassium phosphate buffer (pH 6.0 for ST3Gal1 and ST6GalNAc1, pH 7.0 for dC1GalT)) and cultivated at 20 °C with continuous shaking (140 rpm). Methanol (1 mL) was added every 24 hours to the medium. After 72 hours of cultivation, the supernatant was obtained by centrifuging at 11,000 × *g* at 4 °C for 10 minutes. One millilitre of 100 mM phenylmethylsulfonyl fluoride in dimethyl sulfoxide and one tablet of protease inhibitor (complete EDTA free, Roche Diagnostics, Tokyo, Japan) were added to the supernatant. The supernatant was filtered with a glass microfiber filter (GE Healthcare) and stored at −20 °C until purification.

### Purification of dC1GalT

The thawed supernatant (50 mL) was dialysed against binding buffer (20 mM sodium phosphate, 0.5 M sodium chloride, 0.1% Triton X-100, pH 7.4). The dialysed sample was then carefully titrated to pH 7.4 with sodium hydroxide, filtrated with a 0.45 μm filter and loaded on a HisTrap HP column (5 mL, GE Healthcare) equilibrated with binding buffer. After washing the column with 10 column volumes (CV) of binding buffer, the enzyme was eluted with eluting buffer (20 mM sodium phosphate, 0.5 M sodium chloride, 0.5 M imidazole, 0.1% Triton X-100, pH 7.4) using a stepwise gradient (10 CV of 10% eluting buffer, followed by 5 CV of 100% eluting buffer). Each fraction was checked by sodium dodecyl sulfate polyacrylamide gel electrophoresis (SDS-PAGE) and Western blotting to determine the purity (data not shown). The fractions containing dC1GalT were concentrated by ultrafiltration (Amicon Ultra-15 Centrifugal Filter Units, 30,000 NMWL, Merck Millipore, Darmstadt, Germany).

### Purification of ST3Gal1

The thawed supernatant (100 mL) was dialysed against binding buffer (20 mM sodium phosphate, 0.5 M sodium chloride, 0.1% Triton X-100, pH 7.3). The dialysed sample was then carefully titrated to pH 7.3 with sodium hydroxide, filtrated with a 0.45 μm filter and loaded on a HisTrap HP column (5 mL, GE Healthcare) equilibrated in binding buffer. After washing the column with five CV of binding buffer, the enzyme was eluted with eluting buffer (20 mM sodium phosphate, 0.5 M sodium chloride, 0.5 M imidazole, 0.1% Triton X-100, pH 7.3) using a stepwise gradient (five CV of 10% eluting buffer, followed by five CV of 100% eluting buffer). Each fraction was checked by SDS-PAGE and Western blotting to determine the purity (data not shown). The fractions containing ST3Gal1 were concentrated by ultrafiltration (Amicon Ultra-15, 10,000 NMWL, Merck Millipore).

### Purification of ST6GalNAc1

The thawed supernatant (100 mL) was dialysed against binding buffer (20 mM 2-(*N*-morpholino)ethanesulfonic acid, 0.1% Triton-X100, pH 6.3). The dialysed sample was then carefully titrated to pH 6.3 with HCl, filtrated with a 0.45 μm filter and loaded on a HiTrap SP column (5 mL, GE Healthcare) equilibrated in binding buffer. After washing the column with 10 CV of binding buffer, the enzyme was eluted with eluting buffer (20 mM 2-(*N*-morpholino)ethanesulfonic acid, 1 M sodium chloride, 0.1% Triton-X100, pH 6.3) using a stepwise gradient (eight CV of 20% eluting buffer, eight CV of 50% eluting buffer, followed by eight CV of 100% eluting buffer). Each fraction was checked by SDS-PAGE and Western blotting to determine the purity (data not shown). The fractions containing ST6GalNAc1 were concentrated by ultrafiltration (Amicon Ultra-15, 10,000 NMWL, Merck Millipore).

### Enzymatic reaction (small scale for evaluation of reactivity on different substrates)

The reaction was tested in duplicate at 37 °C with a reaction mixture (10 μL) containing 100 mM 3-(*N*-morpholino)propanesulfonic acid (pH 7.3), 10 mM MnCl_2_, 100 μM acceptor glycopeptide, 300 μM sugar nucleotide (UDP-Gal or CMP-Neu5Ac), 1 mM phenylmethylsulfonyl fluoride, complete protease inhibitor (Roche Diagnostics), and started by addition of 2 μL of enzyme solutions. For reactions of ST6GalNAc1 and ST3Gal1, alkaline phosphatase (*E. coli* C75, 0.01 U, Takara Bio, Shiga, Japan) was also added. At three, six and 18 hours of reaction, 2 μL of the reaction mixture was collected and heated at 95 °C for five minutes to terminate the reaction. The sample was dissolved with 10 μL of water and applied for HPLC analysis. To monitor the time course of the reaction, the enzyme concentration was optimised for each enzyme.

### Analytical HPLC of enzymatic reactions

HPLC analysis was performed on a Shimadzu SCL-10A VP apparatus (Kyoto, Japan) equipped with a UV detector SPD -10AV (set at 220 nm), an autosampler SIL-10ADVP, LC-10 ADVP pumps, CTO-10AC VP column oven (set at 40 °C) and a RF10 AXL Cell temp controller (set at 25 °C). Eluents for dC1GalT reactions were 0.05% trifluoroacetic acid in water (buffer A) and 0.05% trifluoroacetic acid in acetonitrile (buffer B). For ST3Gal1 and ST6GalNAc1, 10 mM trimethylamine acetate in water (buffer A) and 10 mM trimethylamine acetate in acetonitrile (buffer B) were used. Standard conditions comprised a flow rate of 1.0 mL/minute eluting with 10% B to 30% B in 20 minutes on InertSustain® AQ-C18 column (Ø 4.6 mm × 250 mm, 5 μm, GL Sciences, Tokyo, Japan).

### Enzymatic synthesis of MUC1 STn-S_19_

The reaction was performed at 37 °C in a reaction mixture (200 μL) containing 100 mM 3-(*N*-morpholino)propanesulfonic acid (pH 7.3), 10 mM MnCl_2_, 350 μM MUC1 T-Ser_19_, 1 mM CMP-Neu5Ac, 1 mM phenylmethylsulfonyl fluoride, complete protease inhibitor (Roche Diagnostics), alkaline phosphatase *E. coli* C75 (2 U, Takara Bio) and 23 μU ST6GalNAc1. After 24 hours of incubation, ST6GalNAc1 (15 μU, 70 μL) and CMP-Neu5Ac (100 nmol, 1 μL) were added to the reaction mixture. After further 24 hours of incubation at 37 °C, the reaction mixture was heated at 95 °C for five minutes and was lyophilised. The resulting residue was dissolved with 150 μL of water and filtered over a 0.22 μm filter. The filtrate was subjected to HPLC in the same condition with analytical HPLC as described above. The α2,6-sialylated product was isolated and dried by lyophilisation. The residue was dissolved with 50 μL of water, and 6 μL of Glycobuffer 4 (10×, New England BioLabs) was added to the mixture, followed by 15 μL of β1-3,4 galactosidase (120 U, from bovine testis, New England BioLabs). The reaction mixture was incubated for 18 hours at 37 °C, filtered over a 0.22 μm filter, and subjected to HPLC purification as same as after sialylation. The purified fraction was lyophilised and gave MUC1 STn-Ser_19_ (16.3 nmol, 23% yield). MALDI-TOF MS: C_128_H_201_N_36_O_48_ [M + H]^+^ calculated (m/z) 3010.44, found (m/z) 3010.42.

### ELISA to determine the binding specificities of 13 anti-MUC1 mAbs to a panel of 20 glycopeptides

Glycopeptides were diluted in 0.05 M NaHCO_3_, pH 9.6, coated at a concentration of 1 μg/mL in a volume of 100 μL/well to 96-well MaxiSorp plates (Nunc, Roskilde, Denmark) and incubated at 4 °C overnight. Plates were washed three times with PBS containing 0.05% (v/v) Tween (PBST) using a 405TS Miroplate Washer (BioTek, Winooski, VT) and then blocked with a volume of 200 μL/well of 3% (w/v) BSA/PBS at room temperature for two hours. Plates were washed three times with PBST and then incubated with primary antibodies or isotype control antibodies diluted in 1% (w/v) BSA/PBS to a concentration of 0.25 μg/mL (Ma695 0.5 μg/mL) and using a volume of 50 μL/well at room temperature for two hours. Plates were washed three times with PBST and then incubated with peroxidase-conjugated AffiniPure goat anti-mouse IgG (H + L) (Jackson ImmunoResearch Laboratories, West Grove, PA), diluted 1:10000 in 1% (w/v) BSA/PBS using a volume of 100 μL/well at room temperature for one hour. After plates were washed six times with PBST, Super AquaBlue ELISA Substrate (Thermo Fisher Scientific, Waltham, MA) was added using a volume of 100 μL/well and incubated at room temperature for 35 minutes to visualise antibody binding. Absorbance was measured at a wavelength of 405/492 nm by a Multiscan FC spectrophotometer (Thermo Fisher Scientific). Values were generated in duplicates and repeated at least two times.

### Statistical analysis

Correlation analysis of the antibody binding data was performed by using WGCNA package^31^ in Microsoft R open 3.5.1 (https://mran.microsoft.com/open). Pearson’s correlation coefficient between Ab-peptide binding intensities was calculated by using “cor” function in Microsoft R open 3.5.1. Statistical significance of the correlation was calculated by using Fisher’s exact test (two-sided) which is integrated in “corPvalueFisher” function in WGCNA package. Data was visualised by using “labeledHeatmap” function in WGCNA package in Microsoft R open 3.5.1.

## Supplementary information

Supplementary information and figures

## Data Availability

All data generated or analysed during this study are included in this published article (and its Supplementary Information files).

## References

[1] Gendler SJ, Spicer AP (1995). Epithelial mucin genes. Annu Rev Physiol.

[2] Hanisch FG, Ninkovic T (2006). Immunology of O-glycosylated proteins: approaches to the design of a MUC1 glycopeptide-based tumor vaccine. Curr Protein Pept Sci.

[3] Li X (2018). Clinicopathological and Prognostic Significance of Cancer Antigen 15-3 and Carcinoembryonic Antigen in Breast Cancer: A Meta-Analysis including 12,993 Patients. Dis Markers.

[4] Kohno N (1999). Serum marker KL-6/MUC1 for the diagnosis and management of interstitial pneumonitis. J Med Invest.

[5] Karsten U (1998). Enhanced binding of antibodies to the DTR motif of MUC1 tandem repeat peptide is mediated by site-specific glycosylation. Cancer Res.

[6] Karsten U, Serttas N, Paulsen H, Danielczyk A, Goletz S (2004). Binding patterns of DTR-specific antibodies reveal a glycosylation-conditioned tumor-specific epitope of the epithelial mucin (MUC1). Glycobiology.

[7] Tarp MA (2007). Identification of a novel cancer-specific immunodominant glycopeptide epitope in the MUC1 tandem repeat. Glycobiology.

[8] Ohyabu N (2009). An essential epitope of anti-MUC1 monoclonal antibody KL-6 revealed by focused glycopeptide library. J Am Chem Soc.

[9] Rangappa S (2016). Effects of the multiple O-glycosylation states on antibody recognition of the immunodominant motif in MUC1 extracellular tandem repeats. Med. Chem. Commun..

[10] Zhou Dapeng, Xu Lan, Huang Wei, Tonn Torsten (2018). Epitopes of MUC1 Tandem Repeats in Cancer as Revealed by Antibody Crystallography: Toward Glycopeptide Signature-Guided Therapy. Molecules.

[11] Matsushita T, Hinou H, Kurogochi M, Shimizu H, Nishimura S (2005). Rapid microwave-assisted solid-phase glycopeptide synthesis. Org Lett.

[12] Blixt O, Allin K, Pereira L, Datta A, Paulson JC (2002). Efficient chemoenzymatic synthesis of O-linked sialyl oligosaccharides. J Am Chem Soc.

[13] Kono M (2000). Redefined substrate specificity of ST6GalNAc II: a second candidate sialyl-Tn synthase. Biochem Biophys Res Commun.

[14] Takeuchi H (2002). The epitope recognized by the unique anti-MUC1 monoclonal antibody MY.1E12 involves sialyl alpha 2-3galactosyl beta 1-3N-acetylgalactosaminide linked to a distinct threonine residue in the MUC1 tandem repeat. J Immunol Methods.

[15] Bennett EP (2012). Control of mucin-type O-glycosylation: a classification of the polypeptide GalNAc-transferase gene family. Glycobiology.

[16] Schuman J, Campbell AP, Koganty RR, Longenecker BM (2003). Probing the conformational and dynamical effects of O-glycosylation within the immunodominant region of a MUC1 peptide tumor antigen. J Pept Res.

[17] Coelho H (2015). The Quest for Anticancer Vaccines: Deciphering the Fine-Epitope Specificity of Cancer-Related Monoclonal Antibodies by Combining Microarray Screening and Saturation Transfer Difference NMR. J Am Chem Soc.

[18] Martinez-Saez N (2015). Deciphering the Non-Equivalence of Serine and Threonine O-Glycosylation Points: Implications for Molecular Recognition of the Tn Antigen by an anti-MUC1 Antibody. Angew Chem Int Ed Engl.

[19] Price MR (1998). Summary report on the ISOBM TD-4 Workshop: analysis of 56 monoclonal antibodies against the MUC1 mucin. Tumour Biol.

[20] Bennett EP (1998). Cloning of a human UDP-N-acetyl-alpha-D-Galactosamine:polypeptide N-acetylgalactosaminyltransferase that complements other GalNAc-transferases in complete O-glycosylation of the MUC1 tandem repeat. J Biol Chem.

[21] Olson FJ, Backstrom M, Karlsson H, Burchell J, Hansson GC (2005). A MUC1 tandem repeat reporter protein produced in CHO-K1 cells has sialylated core 1 O-glycans and becomes more densely glycosylated if coexpressed with polypeptide-GalNAc-T4 transferase. Glycobiology.

[22] Hanisch FG (1999). Dynamic epigenetic regulation of initial O-glycosylation by UDP-N-Acetylgalactosamine:Peptide N-acetylgalactosaminyltransferases. site-specific glycosylation of MUC1 repeat peptide influences the substrate qualities at adjacent or distant Ser/Thr positions. J Biol Chem.

[23] Wandall HH (1997). Substrate specificities of three members of the human UDP-N-acetyl-alpha-D-galactosamine:Polypeptide N-acetylgalactosaminyltransferase family, GalNAc-T1, -T2, and -T3. J Biol Chem.

[24] Sorensen AL (2006). Chemoenzymatically synthesized multimeric Tn/STn MUC1 glycopeptides elicit cancer-specific anti-MUC1 antibody responses and override tolerance. Glycobiology.

[25] Posey AD (2016). Engineered CAR T Cells Targeting the Cancer-Associated Tn-Glycoform of the Membrane Mucin MUC1 Control Adenocarcinoma. Immunity.

[26] Wilkie S (2008). Retargeting of human T cells to tumor-associated MUC1: the evolution of a chimeric antigen receptor. J Immunol.

[27] Yamamoto M, Bhavanandan VP, Nakamori S, Irimura T (1996). A novel monoclonal antibody specific for sialylated MUC1 mucin. Jpn J Cancer Res.

[28] Kuroda K (2007). Antibody expression in protease-deficient strains of the methylotrophic yeast Ogataea minuta. FEMS Yeast Res.

[29] Murakami S (2013). Identification and characterization of endo-beta-N-acetylglucosaminidase from methylotrophic yeast Ogataea minuta. Glycobiology.

[30] Fujii Y (2014). PA tag: a versatile protein tagging system using a super high affinity antibody against a dodecapeptide derived from human podoplanin. Protein Expr Purif.

[31] Langfelder P, Horvath S (2008). WGCNA: an R package for weighted correlation network analysis. BMC Bioinformatics.

