# Supplementary material for: Products of Chemoenzymatic Synthesis Representing MUC1 Tandem Repeat Unit with T-, ST- or STn-antigen Revealed Distinct Specificities of Anti-MUC1 Antibodies
Source: Sci Rep. 2019 Nov 12;9:16641. doi: 10.1038/s41598-019-53052-1 (PMC6851390; doi:10.1038/s41598-019-53052-1)
Supplement: Supplementary file 1 — Supplementary information and figures [file 41598_2019_53052_MOESM1_ESM.docx]

Scientific Reports

**Products of Chemoenzymatic Synthesis Representing MUC1 Tandem Repeat Unit with T-, ST- or STn-antigen Revealed Distinct Specificities of Anti-MUC1 Antibodies**

*Yayoi Yoshimura, Kaori Denda-Nagai, Yoshie Takahashi, Izuru Nagashima, Hiroki Shimizu, Toshimitsu Kishimoto, Miki Noji, Shigeyuki Shichino, Yasunori Chiba and Tatsuro Irimura*

**Supplementary information and supplementary figures**

**Preparative-scale enzymatic synthesis of MUC1 T-Thr_8_.** The reaction was performed at 37°C in a reaction mixture (368 μL) containing 100 mM 3-(N-morpholino)propanesulfonic acid (MOPS) (pH 7.3), 10 mM MnCl_2_, 640 μM MUC1 Tn-Thr_8_ glycopeptide, 1.6 mM UDP-Gal, 1 mM phenylmethylsulfonyl fluoride (PMSF), complete protease inhibitor (Roche Diagnostics), and 800 μU C1GalT. After four hours since starting the reaction, the reaction mixture was filtrated using a 0.22 μm filter. The filtrate was subjected to high pressure liquid chromatography (HPLC) in the same condition with analytical HPLC as described in the Material and Methods section. The purified fraction was lyophilised and gave MUC1 ST-Thr_8_ (188 nmol, 80% yield). Matrix-assisted laser desorption-time of flight mass spectrometry (MALDI-TOF MS): C_123_H_149_N_35_O_45_ [M+H]^+^ calculated (m/z) 2881.40, found (m/z) 2881.31.

**Preparative-scale enzymatic synthesis of MUC1 T-Ser_9_,** **MUC1 T-Thr_13_, MUC1 T-Ser_19_ or MUC1 T-Thr_20_.** The reaction was performed at 37°C in a reaction mixture (308 μL) containing 100 mM MOPS (pH 7.3), 10 mM MnCl_2_, 650 μM corresponding MUC1 Tn glycopeptide, 1.6 mM UDP-Gal, 1 mM PMSF, complete protease inhibitor (Roche Diagnostics), and 600 μU C1GalT. After four to five hours since starting the reaction, the reaction mixture was filtrated using a 0.22 μm filter. The filtrate was subjected to HPLC in the same condition with analytical HPLC as described in the Material and Methods section. The purified fraction was lyophilised and gave each MUC1 glycopeptide carrying T-antigen: MUC1 T-Ser_9_ (164.2 nmol, 82% yield); MUC1 T-Thr_13_ (190.0 nmol, 95% yield); MUC1 T-Ser_19_ (161.8 nmol, 81%); MUC1 T-Thr_20_ (161.2 nmol, 81%). MALDI-TOF MS: C_123_H_194_N_35_O_45_ [M+H]^+^ calculated (m/z) 2881.40, found (m/z) MUC1 T-Ser_9_ 2881.32; MUC1 T-Thr_13_ 2881.30; MUC1 T-Ser_19_ 2881.25; MUC1 T-Thr_20_ 2881.19.

**Preparative-scale enzymatic synthesis of MUC1 ST-Thr_8_.** The reaction was performed at 37°C in a reaction mixture (150 μL) containing 100 mM MOPS (pH 7.3), 10 mM MnCl_2_, 329 μM MUC1 T-Thr_8_, 1.6 mM CMP-Neu5Ac, 1 mM PMSF, complete protease inhibitor (Roche Diagnostics), and 752 μU ST3Gal1. After four hours since starting the reaction, the reaction mixture was filtrated using a 0.22 μm filter. The filtrate was subjected to HPLC in the same condition with analytical HPLC as described in the Material and Methods section. The purified fraction was lyophilised and gave MUC1 ST-Thr_8_ (33.1 nmol, 67% yield). MALDI-TOF MS: C_134_H_211_N_36_O_53_ [M+H]^+^ calculated (m/z) 3172.49, found (m/z) 3172.40.

**Preparative-scale enzymatic synthesis of MUC1 ST-Ser_9_.** The reaction was performed at 37°C in a reaction mixture (180 μL) containing 100 mM MOPS (pH 7.3), 10 mM MnCl_2_, 274 μM MUC1 T-Ser_9_, 1.4 mM CMP-Neu5Ac, 1 mM PMSF, complete protease inhibitor (Roche Diagnostics), and 752 μU ST3Gal1. After four hours since starting the reaction, the reaction mixture was filtrated using a 0.22 μm filter. The filtrate was subjected to HPLC in the same condition with analytical HPLC as described in the Material and Methods section. The purified fraction was lyophilised and gave MUC1 ST-Ser_9_ (34.5 nmol, 70% yield). MALDI-TOF MS: C_134_H_211_N_36_O_53_ [M+H]^+^ calculated (m/z) 3172.49, found (m/z) 3172.40.

**Preparative-scale enzymatic synthesis of MUC1 ST-Thr_13_.** The reaction was performed at 37°C in a reaction mixture (150 μL) containing 100 mM MOPS (pH 7.3), 10 mM MnCl_2_, 327 μM MUC1 T-Thr_13_, 1.7 mM CMP-Neu5Ac, 1 mM PMSF, complete protease inhibitor (Roche Diagnostics), and 960 μU ST3Gal1. After eight hours since starting the reaction, the reaction mixture was filtrated using a 0.22 μm filter. The filtrate was subjected to HPLC in the same condition with analytical HPLC as described in the Material and Methods section. The purified fraction was lyophilised and gave MUC1 ST-Thr_13_ (47.6 nmol, 97% yield). MALDI-TOF MS: C_134_H_211_N_36_O_53_ [M+H]^+^ calculated (m/z) 3172.49, found (m/z) 3172.38.

**Preparative-scale enzymatic synthesis of MUC1 ST-Ser_19_.** The reaction was performed at 37°C in a reaction mixture (187 μL) containing 100 mM MOPS (pH 7.3), 10 mM MnCl_2_, 261 μM MUC1 T-Ser_19_, 1.3 mM CMP-Neu5Ac, 1 mM PMSF, complete protease inhibitor (Roche Diagnostics), and 940 μU ST3Gal1. After four hours since starting the reaction, the reaction mixture was filtrated using a 0.22 μm filter. The filtrate was subjected to HPLC in the same condition with analytical HPLC as described in the Material and Methods section. The purified fraction was lyophilised and gave MUC1 ST-Ser_19_ (38.6 nmol, 79% yield). MALDI-TOF MS: C_134_H_211_N_36_O_53_ [M+H]^+^ calculated (m/z) 3172.49, found (m/z) 3172.35.

**Preparative-scale enzymatic synthesis of MUC1 ST-Thr_20_.** The reaction was performed at 37°C in a reaction mixture (140 μL) containing 100 mM MOPS (pH 7.3), 10 mM MnCl_2_, 353 μM MUC1 T-Thr_20_, 1.8 mM CMP-Neu5Ac, 1 mM PMSF, complete protease inhibitor (Roche Diagnostics), and 940 μU ST3Gal1. After four hours since starting the reaction, the reaction mixture was filtrated using a 0.22 μm filter. The filtrate was subjected to HPLC in the same condition with analytical HPLC as described in the Material and Methods section. The purified fraction was lyophilised and gave MUC1 ST-Thr_20_ (47.9 nmol, 97% yield). MALDI-TOF MS: C_134_H_211_N_36_O_53_ [M+H]^+^ calculated (m/z) 3172.49, found (m/z) 3172.29.

**Preparative-scale enzymatic synthesis of MUC1 STn-Thr_8_.** The reaction was performed at 37°C in a reaction mixture (284 μL) containing 100 mM MOPS (pH 7.3), 10 mM MnCl_2_, 350 μM MUC1 Tn-Thr_8_, 1 mM CMP-Neu5Ac, 1 mM PMSF, complete protease inhibitor (Roche Diagnostics), alkaline phosphatase *E. coli* C75 (2 U, Takara Bio) and 46 μU ST6GalNAc1. After 18 hours since starting the reaction, a mixture (100 μL) containing 1 mM CMP-Neu5Ac and 41 μU ST6GalNAc1 was added to the reaction mixture. After further incubation for seven hours, a mixture (80 μL) containing 100 mM MOPS (pH 7.3), 10 mM MnCl_2_, 1 mM CMP-Neu5Ac, alkaline phosphatase *E. coli* C75 (1.5 U) and 23 μU ST6GalNAc1 was added to the reaction mixture. After further incubation for 18 hours (total reaction time 43 hours), the reaction mixture was filtrated using a 0.22 μm filter. The filtrate was subjected to HPLC in the same condition with analytical HPLC as described in the Material and Methods section. The purified fraction was lyophilised and gave MUC1 STn-Thr_8_ (41.7 nmol, 42% yield). MALDI-TOF MS: C_128_H_201_N_36_O_48_ [M+H]^+^ calculated (m/z) 3010.44, found (m/z) 3010.57._._

**Preparative-scale enzymatic synthesis of MUC1 STn-Thr_13_.** The reaction was performed at 37°C in a reaction mixture (140 μL) containing 100 mM MOPS (pH 7.3), 10 mM MnCl_2_, 353 μM MUC1 Tn-Thr_13_, 1.1 mM CMP-Neu5Ac, 1 mM PMSF, complete protease inhibitor (Roche Diagnostics), alkaline phosphatase *E. coli* C75 (2 U, Takara Bio) and 23 μU ST6GalNAc1. After 18 hours since starting the reaction, the reaction mixture was filtrated using a 0.22 μm filter. The filtrate was subjected to HPLC in the same condition with analytical HPLC as described in Material and Methods section. The purified fraction was lyophilised and gave MUC1 STn-Thr_13_ (28.7 nmol, 58% yield). MALDI-TOF MS: C_128_H_201_N_36_O_48_ [M+H]^+^ calculated (m/z) 3010.44, found (m/z) 3010.45._._

**Preparative-scale enzymatic synthesis of MUC1 STn-Thr_20_.** The reaction was performed at 37°C in a reaction mixture (184 μL) containing 100 mM MOPS (pH 7.3), 10 mM MnCl_2_, 327 μM MUC1 Tn-Thr_20_, 1 mM CMP-Neu5Ac, 1 mM PMSF, complete protease inhibitor (Roche Diagnostics), alkaline phosphatase *E. coli* C75 (2 U, Takara Bio) and 32 μU ST6GalNAc1. After 18 hours since starting the reaction, a mixture (80 μL) containing 1 mM CMP-Neu5Ac and 32 μU ST6GalNAc1 was added to the reaction mixture. After further incubation for seven hours, a mixture (30 μL) containing 100 mM MOPS (pH 7.3), 10 mM MnCl_2_, 1 mM CMP-Neu5Ac, alkaline phosphatase *E. coli* C75 (1.5 U) and 5 μU ST6GalNAc1 was added to the reaction mixture. After further incubation for 18 hours (total reaction time 43 hours), the reaction mixture was filtrated using a 0.22 μm filter. The filtrate was subjected to HPLC in the same condition with analytical HPLC as described in the Material and Methods section. The purified fraction was lyophilised and gave MUC1 STn-Thr_20_ (35.5 nmol, 59% yield). MALDI-TOF MS: C_128_H_201_N_36_O_48_ [M+H]^+^ calculated (m/z) 3010.44, found (m/z) 3010.36.

**Preparative-scale enzymatic synthesis of MUC1 STn-Ser_9_.** The reaction was performed at 37°C in a reaction mixture (200 μL) containing 100 mM MOPS (pH 7.3), 10 mM MnCl_2_, 392 μM MUC1 T-Ser_9_, 1 mM CMP-Neu5Ac, 1 mM PMSF, complete protease inhibitor (Roche Diagnostics), alkaline phosphatase *E. coli* C75 (2 U, Takara Bio) and 23 μU ST6GalNAc1. After 18 hours incubation, a mixture (30 μL) containing 1 mM CMP-Neu5Ac and 14 μU ST6GalNAc1 was added to the reaction mixture. After further 18 hours incubation at 37°C (total reaction time 36 hours), the reaction mixture was heated at 95°C for five minutes and lyophilised. The resulting residue was dissolved with 150 μL of H_2_O and filtered using a 0.22 μm filter. The filtrate was subjected to HPLC in the same condition with analytical HPLC as described above. The α2,6-sialylated product was isolated and dried by lyophilisation. The residue was dissolved with 50 μL of water, and 6 μL of Glycobuffer 4 (10×, New England BioLabs) was added to the mixture, followed by 15 μL of β1-3,4 galactosidase (120 U, from bovine testis, New England BioLabs). The reaction mixture was incubated for 18 hours at 37^o^C, filtered using a 0.22 μm filter, and subjected to HPLC purification in the same way as after sialylation. The purified fraction was lyophilised and gave MUC1 STn-Ser_9_ (18.8 nmol, 24% yield). MALDI-TOF MS: C_128_H_201_N_36_O_48_ [M+H]^+^ calculated (m/z) 3010.44, found (m/z) 3010.45._._

**Sample preparation for sodium dodecyl sulphate-polyacrylamide gel electrophoresis (SDS-PAGE).** Each fractionated sample (45μL) after purification was mixed with 5 μL of 10× glycoprotein denaturing buffer (New England Biolabs, Ipswich, MA), heated at 100°C for 10 minutes, and then cooled down to room temperature. Five microliter of 1 M sodium acetate buffer (pH 5.0) was added to the denatured protein mixture, followed by 0.5 μL of Endo Hf (50 U, New England Biolabs). After incubation at 37°C for more than one hour, the mixture was combined with 10 μL of 6 × SDS buffer (350 mM Tris-HCl, 10% SDS, 600 mM dithiothreitol, 0.012% bromophenol blue and 30% glycerol) and heated at 95°C for five minutes. Each 20 μL of the resulting sample was subjected to SDS-PAGE.

**Western blot to check the purity of purified glycosyltransferases dC1GalT, ST3Gal1 and ST6GalNAc1 expressed by *O. minuta.*** Proteins were electrophoresed under reducing conditions in 10% SDS-PAGE with molecular weight markers (Bio-Rad, Hercules, CA) and subsequently transferred to PVDF membranes. The membranes were blocked by Tris-buffered saline with 0.05% Tween 20 (TBS-T) containing 5% skimmed milk powder for one hour, followed by incubation with peroxidase-conjugated anti-PA tag, rat mAb (Fujifilm Wako Pure Chemical, 1:5000 dilution in blocking buffer) for one hour at room temperature. Membranes were washed with TBS-T three times. Bands were visualised using Amersham ECL prime Western blotting detection reagent (GE Healthcare) and imaged on LAS-3000 (GE Healthcare).

**Table S1.** Comparison of the conversion rates from Tn to STn of MUC1 glycopeptides by ST6GalNAc1 when Tn-Ser_9_ or Tn-Ser_19_ were substituted to Tn-Thr. Data shown are the means of two independent experiments.

Product Conversion (%)

Substrate after 18 hours incubation

MUC1 Tn-Ser_9_🡒 Thr_9_ 100

_
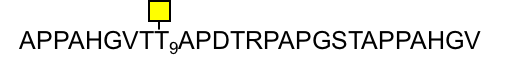
_

MUC1 Tn-Ser_9_ 4


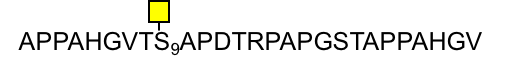


MUC1 Tn-Ser_19_🡒 Thr_19_ 57


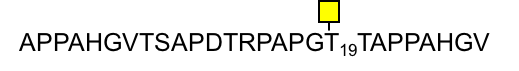


MUC1 Tn-Ser_19_ ND


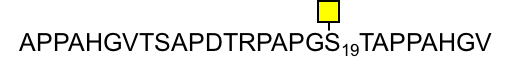


**Figure S1.** HPLC chromatograms of starting materials (upper chromatogram line) and reaction products (lower chromatogram line) from preparative-scale enzymatic synthesis of MUC1 glycopeptides carrying T-antigen using the dC1GalT enzyme. (**a**) Synthesis of MUC1 T-Thr_8_ from MUC1 Tn-Thr_8_. (**b**) Synthesis of MUC1 T-Ser_9_ from MUC1 Tn-Ser_9_. (**c**) Synthesis of MUC1 T-Thr_13_ from MUC1 Tn-Thr_13_. (**d**) Synthesis of MUC1 T-Ser_19_ from MUC1 Tn-Ser_19_. (**e**) Synthesis of MUC1 T-Thr_20_ from MUC1 Tn-Thr_20_.


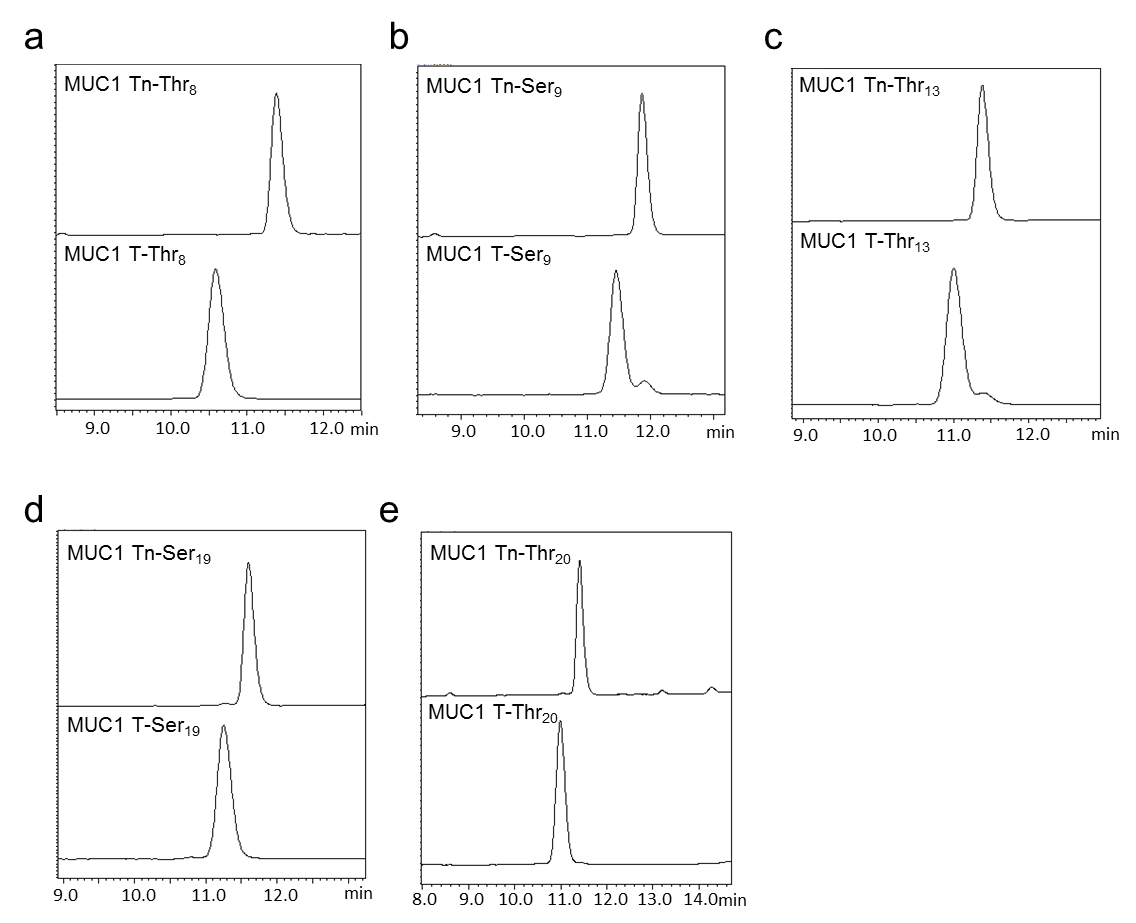


**Figure S2.** HPLC chromatograms of starting materials (upper chromatogram line) and reaction products (lower chromatogram line) from preparative-scale enzymatic synthesis of MUC1 glycopeptides carrying ST-antigen using the ST3Gal1 enzyme. (**a**) Synthesis of MUC1 ST-Thr_8_ from MUC1 T-Thr_8_. (**b**) Synthesis of MUC1 ST-Ser_9_ from MUC1 T-Ser_9_. (**c**) Synthesis of MUC1 ST-Thr_13_ from MUC1 T-Thr_13_. (**d**) Synthesis of MUC1 ST-Ser_19_ from MUC1 T-Ser_19_. (**e**) Synthesis of MUC1 ST-Thr_20_ from MUC1 T-Thr_20_.

_
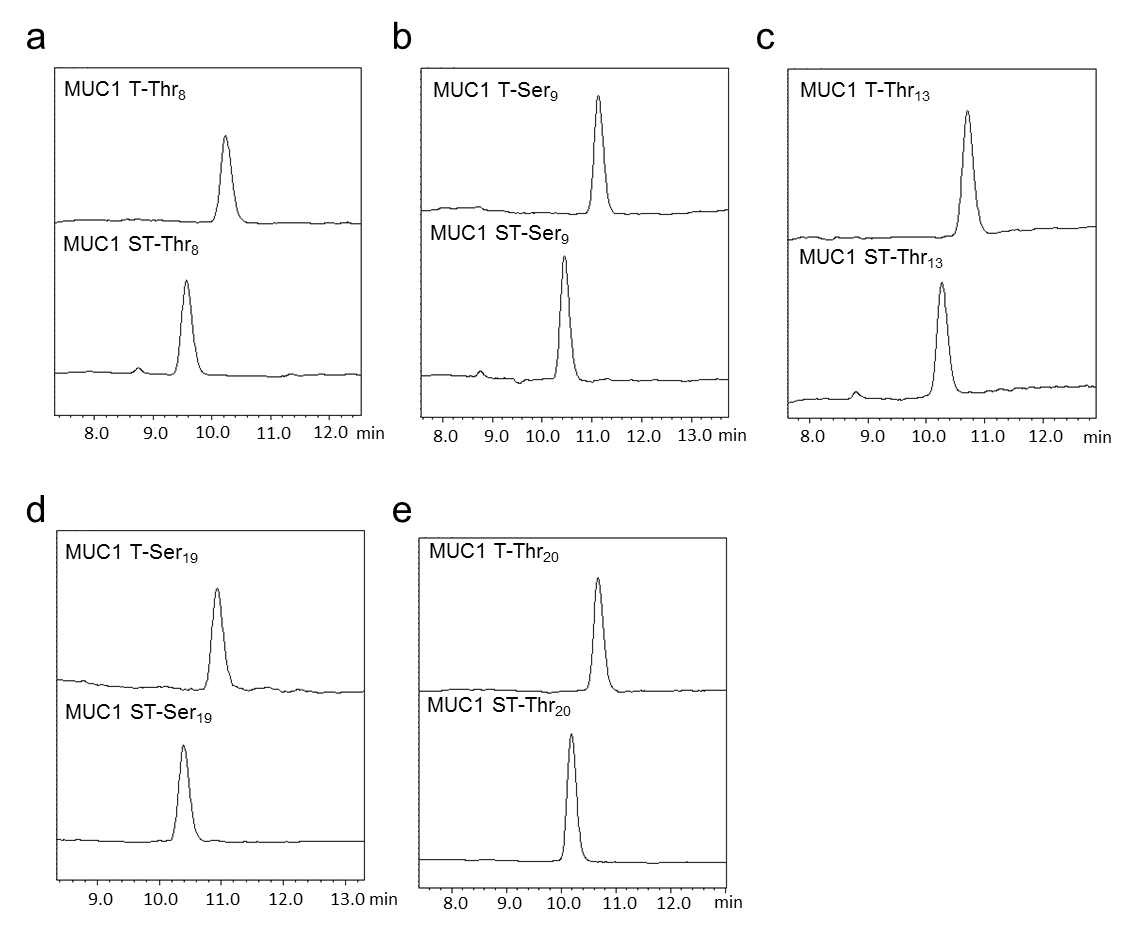
_

**Figure S3.** HPLC chromatograms of starting materials (upper chromatogram line) and reaction products (lower chromatogram line) from preparative-scale enzymatic synthesis of MUC1 glycopeptides carrying STn-antigen using the ST6GalNAc1 enzyme. (**a**) Synthesis of MUC1 STn-Thr_8_ from MUC1 Tn-Thr_8_. (**b**) Synthesis of MUC1 STn-Thr_13_ from MUC1 Tn-Thr_13_. (**c**) Synthesis of MUC1 STn-Thr_20_ from MUC1 Tn-Thr_20_.


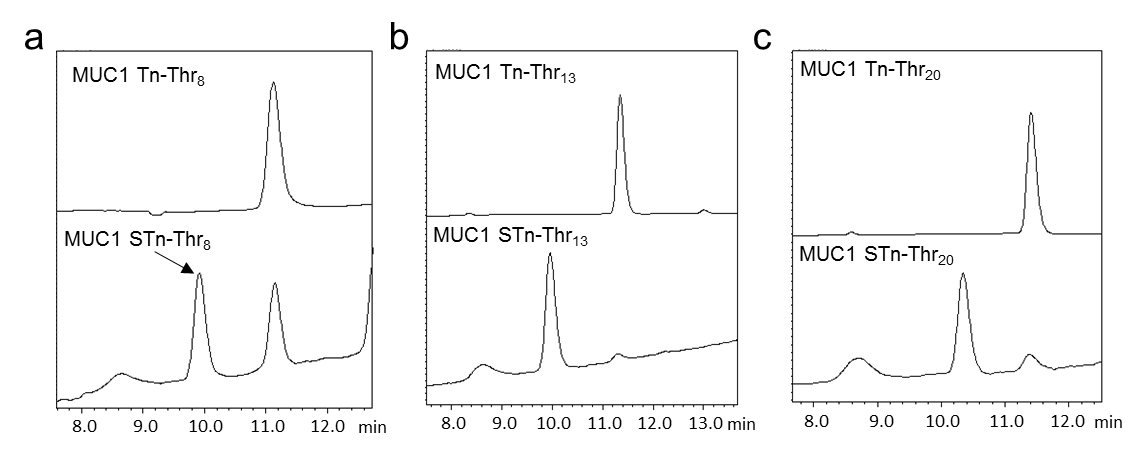


**Figure S4.** Enzymatic synthesis of MUC1 STn-Ser_9_. Within each figure, (i), (ii) and (iii) correspond to MUC1 T-Ser_9_, α2,6-sialylated MUC1 T-Ser_9_ (in reaction mixture) and MUC1 STn-Ser_9_ (in reaction mixture), respectively. (**a**) Schematic representation of the synthesis of MUC1 STn-Ser_9_. The final reaction with β1-3,4 galactosidase was performed on the purified α2,6-sialylated MUC1 T-Ser_9_. (**b**) MALDI-TOF MS spectra of the reaction products from enzymatic synthesis. (**c**) HPLC chromatograms of the reaction products from enzymatic synthesis.


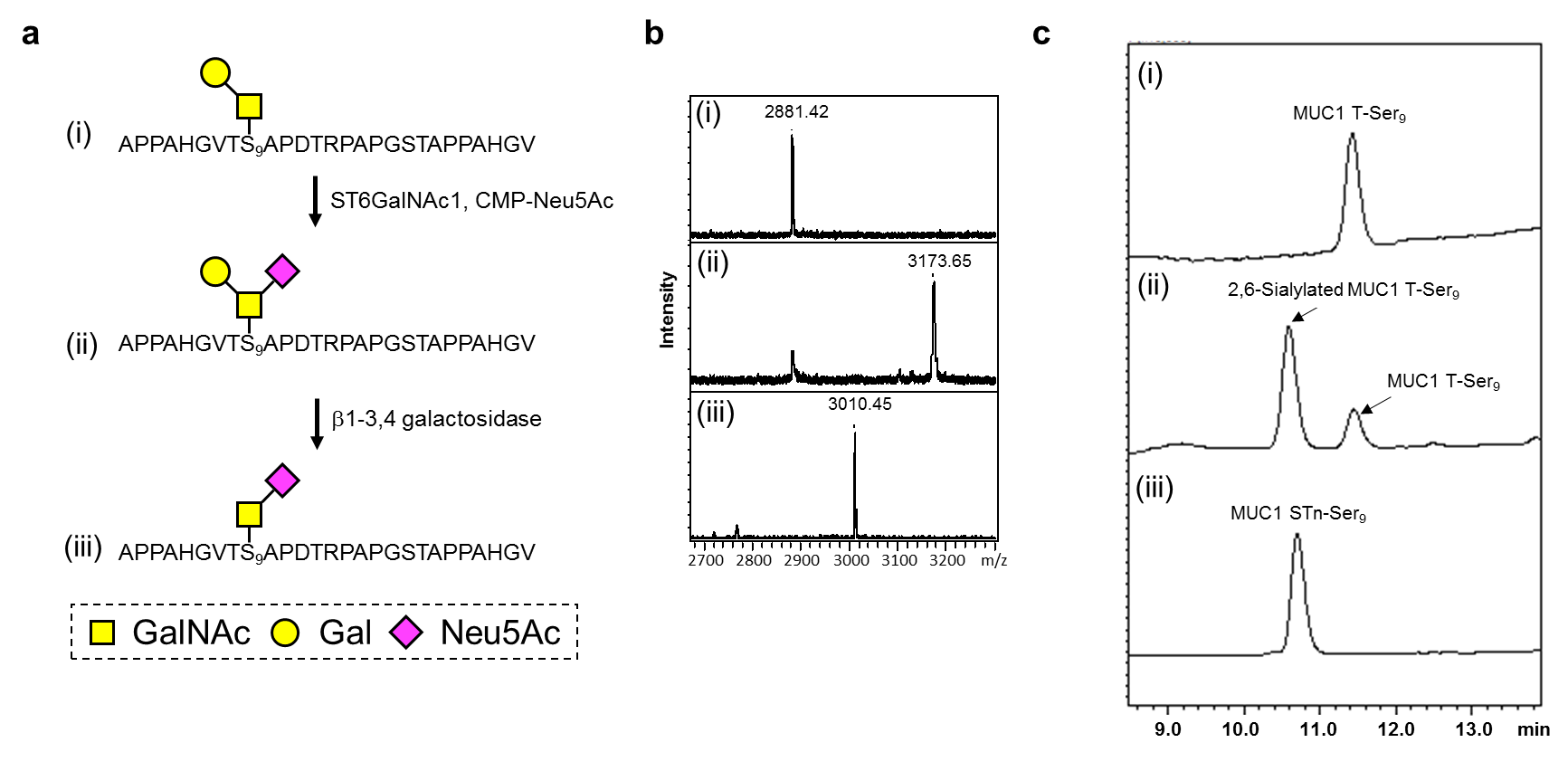


**Figure S5.** Analysis of purified MUC1 peptide and five MUC1 Tn glycopeptides. (**a**) MALDI-TOF MS spectra. (**b**) HPLC chromatograms.


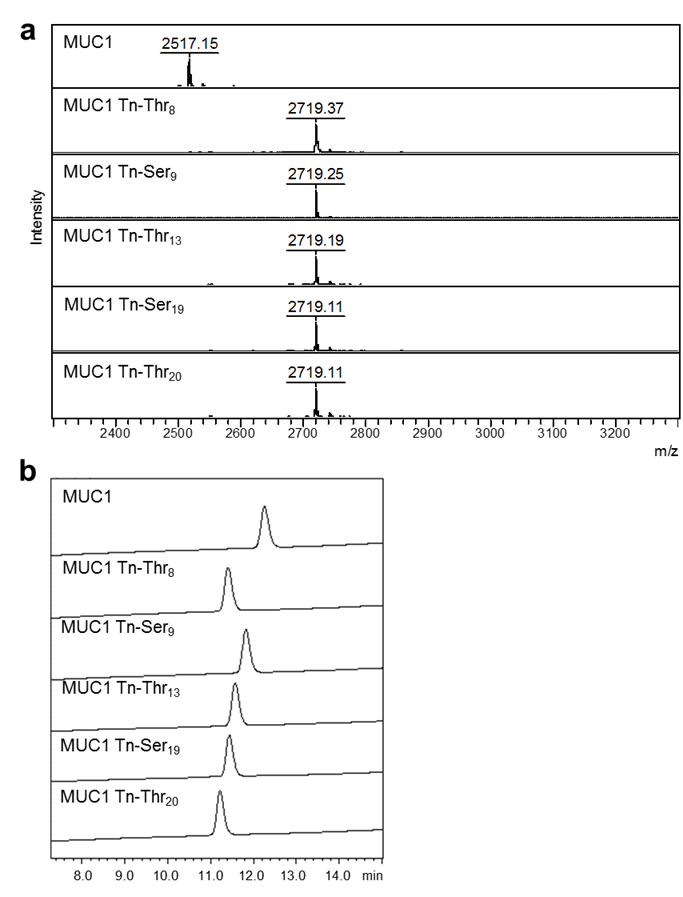


**Figure S6.** Analysis of five purified MUC1 T glycopeptides. (**a**) MALDI-TOF MS spectra. (**b**) HPLC chromatograms.


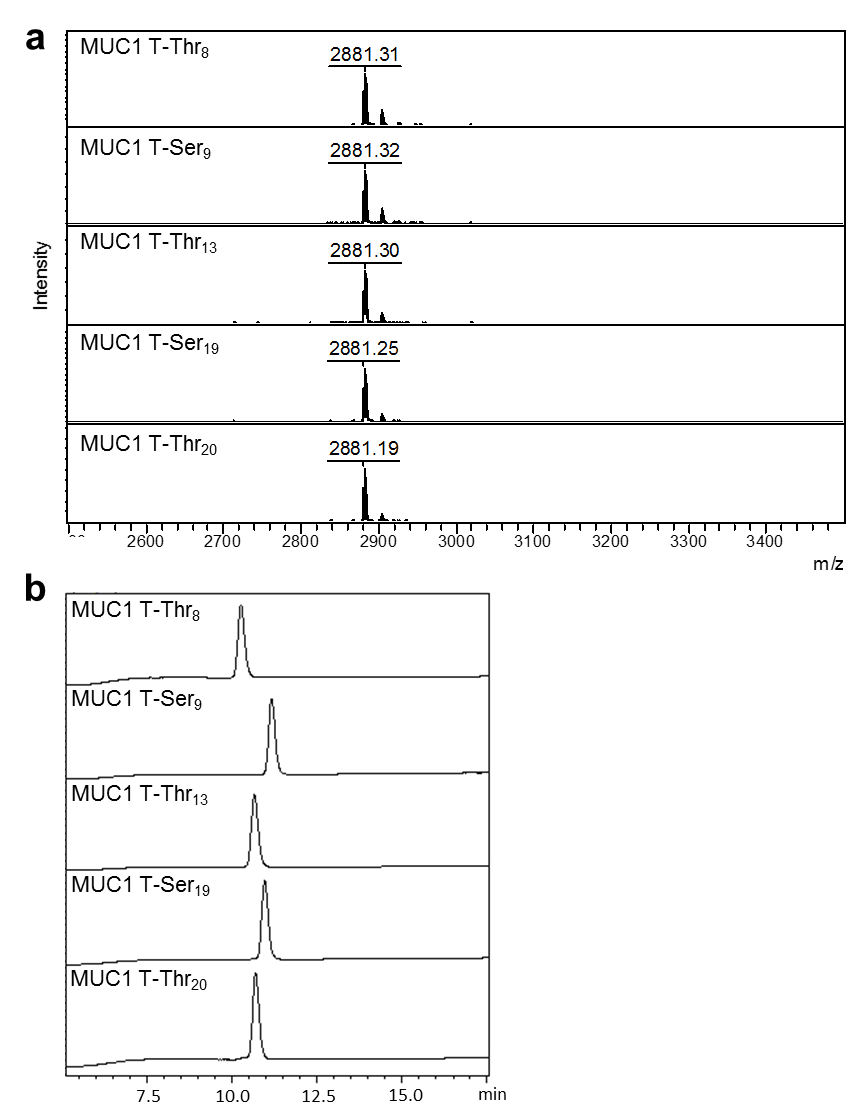


**Figure S7.** Analysis of five purified MUC1 ST glycopeptides. (**a**) MALDI-TOF MS spectra. (**b**) HPLC chromatograms.


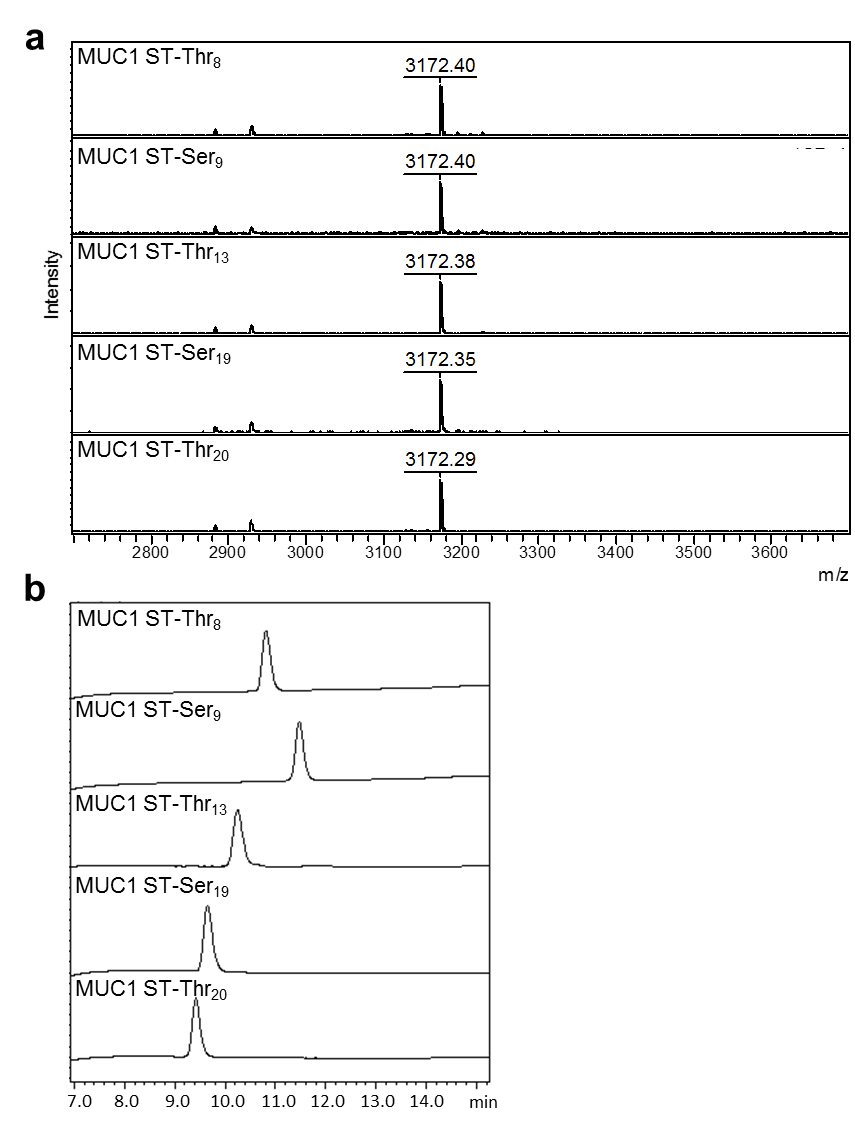


**Figure S8.** Analysis of three purified MUC1 STn glycopeptides. (**a**) MALDI-TOF MS spectra. (**b**) HPLC chromatograms.


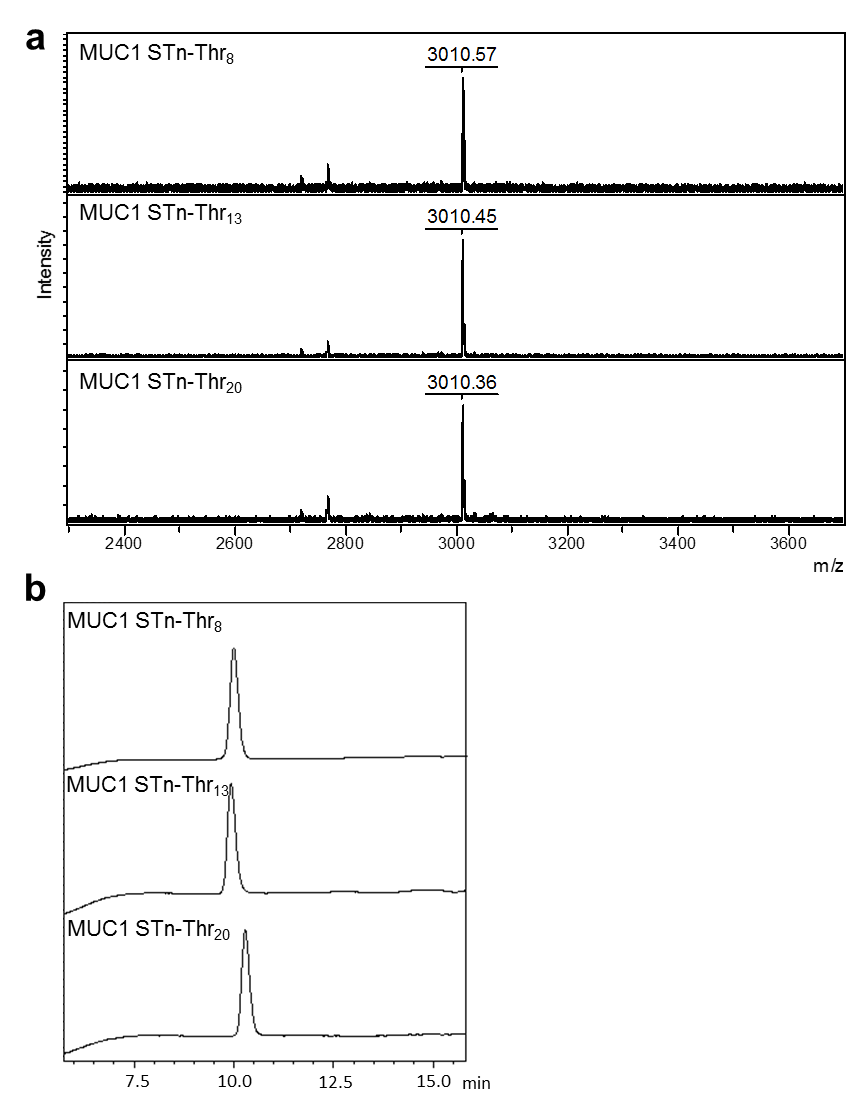


**Figure S9.** Heatmap representation of the correlation of the antibody binding data from Figure 5. Pearson’s correlation coefficients and associated P values (in brackets) are shown.

Correlation analysis revealed very strong positive correlations between the binding patterns of MY.1E12, 115D8, and Ma695 (Group a, Pearson’s r > 0.97) and the binding patterns of SM3 and VU11E2 (Group b, Pearson’s r = 0.83), respectively. The binding pattern of VU4H5 was shown to have a moderate positive correlation (Pearson’s r = 0.44) with the binding pattern of C595. In addition, the binding patterns of Group b antibodies (SM3 and VU11E2) were shown to have a moderate negative correlation (Pearson’s r = -0.54 to -0.6) with the binding patterns of VU4H5 and C595 (Group c). Among Group d, the binding pattern of HMPV was shown to have a moderate positive correlation (Pearson’s r = 0.5) with the binding patterns of E29 and HMFG2.

**
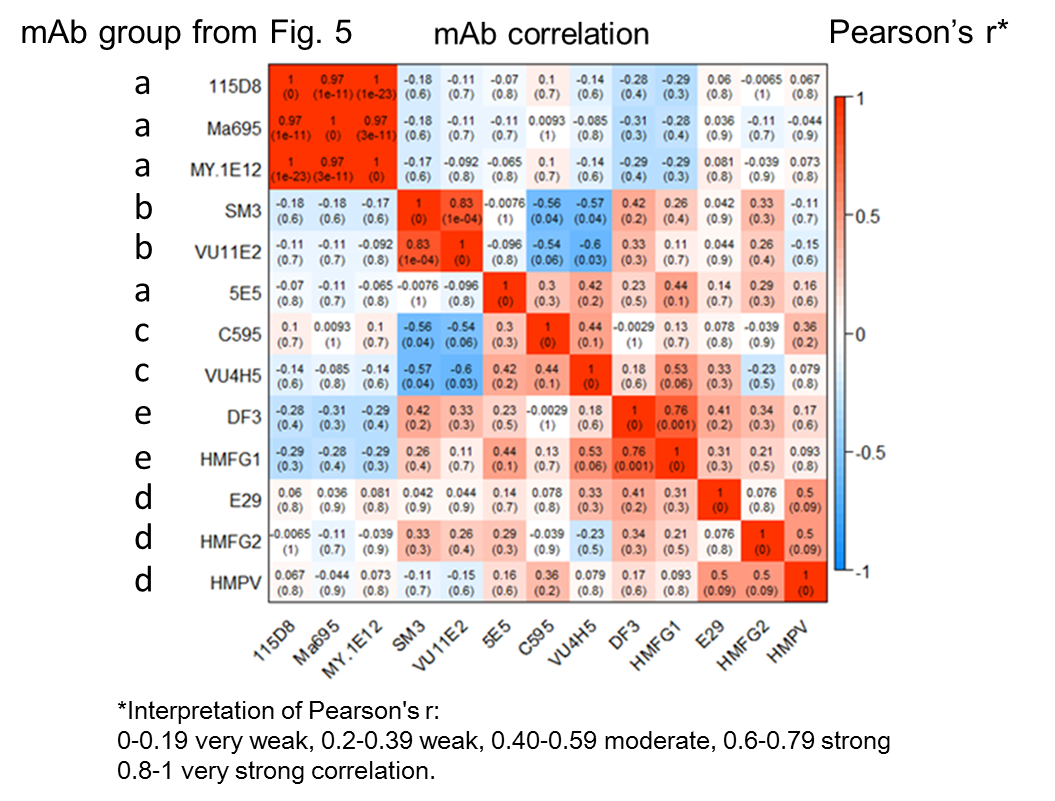
**
